# Specific Commensal Bacterium Critically Regulates Gut Microbiota Osteoimmunomodulatory Actions During Normal Postpubertal Skeletal Growth and Maturation

**DOI:** 10.1002/jbm4.10338

**Published:** 2020-01-30

**Authors:** Jessica D Hathaway‐Schrader, Nicole A Poulides, Matthew D Carson, Joy E Kirkpatrick, Amy J Warner, Brooks A Swanson, Eliza V Taylor, Michael E Chew, Sakamuri V Reddy, Bei Liu, Caroline Westwater, Chad M Novince

**Affiliations:** ^1^ Department of Oral Health Sciences College of Dental Medicine, Medical University of South Carolina Charleston SC USA; ^2^ Department of Pediatrics‐Division of Endocrinology College of Medicine, Medical University of South Carolina Charleston SC USA; ^3^ Department of Drug Discovery & Biomedical Sciences College of Pharmacy, Medical University of South Carolina Charleston SC USA; ^4^ Department of Microbiology and Immunology College of Medicine, Medical University of South Carolina Charleston SC USA

**Keywords:** BONE MODELING/REMODELING, GUT MICROBIOTA, OSTEOCLASTS, OSTEOIMMUNOLOGY

## Abstract

The commensal gut microbiota critically regulates immunomodulatory processes that influence normal skeletal growth and maturation. However, the influence of specific microbes on commensal gut microbiota osteoimmunoregulatory actions is unknown. We have shown previously that the commensal gut microbiota enhances T_H_17/IL17A immune response effects in marrow and liver that have procatabolic/antianabolic actions in the skeleton. Segmented filamentous bacteria (SFB), a specific commensal gut bacterium within phylum Firmicutes, potently induces T_H_17/IL17A‐mediated immunity. The study purpose was to delineate the influence of SFB on commensal gut microbiota immunomodulatory actions regulating normal postpubertal skeletal development. Two murine models were utilized: SFB‐monoassociated mice versus germ‐free (GF) mice and specific‐pathogen‐free (SPF) mice +/− SFB. SFB colonization was validated by 16S rDNA analysis, and SFB‐induced T_H_17/IL17A immunity was confirmed by upregulation of *Il17a* in ileum and IL17A in serum. SFB‐colonized mice had an osteopenic trabecular bone phenotype, which was attributed to SFB actions suppressing osteoblastogenesis and enhancing osteoclastogenesis. Intriguingly, SFB‐colonized mice had increased expression of proinflammatory chemokines and acute‐phase reactants in the liver. Lipocalin‐2 (LCN2), an acute‐phase reactant and antimicrobial peptide, was substantially elevated in the liver and serum of SFB‐colonized mice, which supports the notion that SFB regulation of commensal gut microbiota osteoimmunomodulatory actions are mediated in part through a gut–liver–bone axis. Proinflammatory T_H_17 and T_H_1 cells were increased in liver‐draining lymph nodes of SFB‐colonized mice, which further substantiates that SFB osteoimmune‐response effects may be mediated through the liver. SFB‐induction of *Il17a* in the gut and *Lcn2* in the liver resulted in increased circulating levels of IL17A and LCN2. Recognizing that IL17A and LCN2 support osteoclastogenesis/suppress osteoblastogenesis, SFB actions impairing postpubertal skeletal development appear to be mediated through immunomodulatory effects in both the gut and liver. This research reveals that specific microbes critically impact commensal gut microbiota immunomodulatory actions regulating normal postpubertal skeletal growth and maturation. © 2020 The Authors. *JBMR Plus* published by Wiley Periodicals, Inc. on behalf of American Society for Bone and Mineral Research.

## Introduction

The commensal gut microbiota is the collection of microorganisms colonizing the gastrointestinal (GI) tract in health.^1^, ^2^, ^3^, ^4^ Commensal gut microbes colonize the host following birth, which directs immune development to support a mutualistic relationship between commensal microbiota and the host.^5^, ^6^ Importantly, commensal gut microbiota – immune response effects critically influence the development and homeostasis of host tissues at extra‐GI sites.^1^, ^2^, ^3^, ^4^

The field of osteoimmunology has revealed that innate immune cells, as well as effector T‐cell crosstalk with bone cells, regulate skeletal development and homeostasis.^7^, ^8^ Central to the current report, the commensal gut microbiota has immunoregulatory actions that influence osteoimmune processes in the healthy developing skeleton. Antibiotic disruption of the normal commensal gut microbiota has been shown to drive a proinflammatory immune response state in lymphoid tissues draining the gut, which results in enhanced osteoclastogenesis and impaired bone mass accrual in the postpubertal growing skeleton.^9^ Timely studies employing the germ‐free (GF) mouse model have delineated that the commensal gut microbiota profoundly influences physiologic osteoimmune processes that impact normal skeletal growth and maturation.^10^, ^11^, ^12^, ^13^ Our prior work in 12‐week‐old C57BL/6 specific‐pathogen‐free (SPF) versus GF mice revealed that the commensal gut microbiota enhanced osteoclastogenesis and suppressed osteoblastogenesis, driving bone loss in health.^13^ Intriguingly, the commensal gut microbiota catabolic effects on the skeleton appeared to be through T_H_17/IL17A‐mediated immunity in the bone marrow and liver.^13^ T_H_17 cell‐derived IL17A is a potent pro‐osteoclastic factor that has been shown to promote osteolytic bone loss in autoimmune arthritis,^14^, ^15^ estrogen‐depleted menopausal states,^16^, ^17^ and inflammatory bowel disease.^18^, ^19^

The role of specific microbes in commensal gut microbiota immunomodulatory effects regulating normal skeletal growth and maturation is currently unknown. Of interest, segmented filamentous bacteria (SFB) is a single commensal gut bacterium that potently regulates T_H_17/IL17A‐mediated immunity in mice.^20^, ^21^, ^22^ SFB adheres to epithelial cells to colonize the distal ileum postweaning.^23^, ^24^ Investigations in SPF versus GF mice revealed that SFB is an early gut colonizer, appearing several days postweaning and is prominent throughout development.^21^, ^25^, ^26^, ^27^ Relative to low levels of IL17A expression in GF mice, SFB colonization in SPF mice and SFB‐monoassociated mice dramatically increased T_H_17 cell‐derived IL17A expression in the ileum.^20^, ^21^, ^28^ SFB antigen‐specific T_H_17 cells have been identified in SFB colonized mice,^28^, ^29^, ^30^ whereas nonspecific T_H_17 cells were induced in Peyerʼs patches,^28^, ^29^ isolated lymphoid follicles,^28^, ^29^ and the spleen.^30^ Clinical studies have shown that SFB colonization in humans primarily occurs by 36 months of age, and the presence of SFB in terminal ileal biopsies induced T_H_17 pathway genes.^24^, ^31^

SFB has immunomodulatory actions regulating the development and homeostasis of host tissues, both locally in the gut^20^, ^21^, ^32^ and at extra‐GI sites.^33^, ^34^, ^35^ SFB promotes T_H_17/IL17A‐mediated immunity,^20^, ^21^, ^28^, ^29^, ^30^ which has been shown to aggravate autoimmune conditions distant to the gut. SFB colonization in murine rheumatoid arthritis models exacerbated joint arthropathy through induction of T_H_17/IL17A‐mediated immunity.^33^, ^34^, ^35^ Although SFB has been shown to modulate commensal gut microbiota actions influencing autoimmune arthritis,^33^, ^34^, ^35^ these reports surprisingly did not evaluate osteoimmune processes related to osteoclasts and/or osteoblasts.

The impact of specific microbes on commensal gut microbiota immunomodulatory actions regulating normal skeletal growth and maturation is unknown. The current report carried out studies in GF versus SFB‐monoassociated mice as well as SPF murine models with or without SFB colonization to determine whether specific microbes have the capacity to influence osteoimmune processes in the postpubertal growing skeleton. SFB monoassociation was strategically performed in 5‐week‐old weanling GF mice, because prior reports have shown that SPF mice are spontaneously colonized by SFB several days following weaning.^21^, ^25^, ^26^, ^27^ C57BL/6 female mice typically reach puberty by 5 weeks of age, which is considered the onset of postpubertal skeletal development in the C57BL/6 murine model.^36^, ^37^, ^38^ Mice were sacrificed at 9 weeks of age to evaluate the impact of SFB on postpubertal skeletal development, a robust period of growth and maturation that accounts for approximately 40% of peak bone mass accrual.^39^, ^40^, ^41^, ^42^

Commensal gut microbiota effects on the skeleton have commonly been proposed to occur through a direct gut–bone axis.^43^, ^44^ Our prior work revealed that the commensal gut microbiota profoundly stimulates innate and adaptive immunity in the liver, which led us to postulate that gut microbiota effects on the skeleton are mediated, in part, by a gut–liver–bone axis.^13^ The current report reveals that SFB has pro‐osteoclastic/antiosteoblastic actions impairing postpubertal skeletal development, which appear to be mediated through immunomodulatory effects in both the gut and liver. This research shows that specific microbes critically impact commensal gut microbiota immunomodulatory actions regulating normal postpubertal skeletal growth and maturation.

## Materials and Methods

### GF‐ and SFB‐monoassociated mice

GF C57BL/6 T mice were obtained from Taconic Biosciences (Rensselaer, NY, USA), bred, and maintained in sterile isolators at the Medical University of South Carolina (MUSC; Charleston, SC, USA) Gnotobiotic Animal Core. Room temperature and humidity were maintained within the recommended ranges prescribed by the *Guide for the Care and Use of Laboratory Animals* (8th ed., National Academies Press, Washington, DC; 2011); animals were kept on a 12 hour:12 hour light:dark schedule, and fed sterilized Teklad 8656 diet (Harlan Laboratories, Inc., Indianapolis, IN, USA). Female GF littermates were randomly assigned to be maintained as GF or monoassociated with SFB at 5 weeks of age. GF mice and SFB‐monoassociated mice were group‐housed. SFB‐monoassociated mice were generated by associating GF mice with fresh feces derived from an established SFB‐monoassociated mouse colony. Fresh feces were smeared onto paws, faces, and water bottle lixits of GF mice. Additionally, SFB‐monoassociated mouse‐derived dirty bedding was transferred into cages of GF mice, which was not changed for 1 week following bacterial association. Animals were euthanized at 9 weeks of age. Animal experimentation was approved by the MUSC Institutional Animal Care and Use Committee and carried out in accordance with approved guidelines.

### Murine‐pathogen‐free and excluded‐flora mice

Nine‐week‐old female C57BL/6 T murine‐pathogen‐free (MPF) mice and excluded‐flora [EF] mice were purchased from Taconic Biosciences and euthanized 48 hours following arrival at a SPF vivarium at MUSC. Room temperature and humidity were maintained within the recommended ranges prescribed by the eighth edition of the *Guide for the Care and Use of Laboratory Animals*, animals were kept on a 12 hour:12 hour light:dark schedule, and fed autoclaved NIH‐31 M diet. EF mice and MPF mice were group‐housed. Animal methods were approved by the MUSC Institutional Animal Care and Use Committee and carried out in accordance with approved guidelines.

### Micro‐CT

Tibias were isolated and fixed in 10% phosphate‐buffered formalin and stored in 70% ethanol (EtOH). Specimens were scanned with a Scanco Medical μCT 40 scanner (Scanco Medical, Brüttisellen, Switzerland) with acquisition parameters of X‐ray tube potential at 55 kVp, X‐ray intensity of 145 μA, 200‐ms integration time, and isotropic voxel size of 6 μm^3^. Calibrated 3D images were reconstructed. Trabecular and cortical bone morphology was processed and analyzed using Analyze 12.0 Bone Microarchitecture Analysis software (Analyze Direct, Seattle, WA, USA). Trabecular bone was evaluated by axial CT slices beginning 300 μm distal to the proximal growth plate and extending 1000 μm distally. A fixed threshold of 1750 Hounsfield units (HU) was utilized to discern mineralized tissue. Cortical bone was assessed by transverse CT slices in a 1000‐μm section of the mid‐diaphysis. To discriminate mineralized tissue, a fixed threshold of 2250 HU was used. Data are reported in accordance with standardized nomenclature^45^ as previously described.^9^, ^13^

### Histomorphometry

Tibias were isolated and fixed in 10% phosphate‐buffered formalin for 24 hours at room temperature. Tibias were then decalcified in 14% ethylenediaminetetraacetic acid (EDTA) for 21 days at room temperature and submitted for histological processing. Proximal tibias were paraffin‐embedded, and 5‐μm serial frontal sections were cut. Proximal tibia sections were stained with tartrate‐resistant acid phosphatase (TRAP), and counterstained with a hematoxylin. Histomorphometric analysis of osteoclast cellular outcomes was performed in TRAP‐stained proximal tibia sections; three nonconsecutive sections were scored per animal. TRAP+ multinucleated (three or more nuclei) cells lining bone were scored as osteoclasts. The region of interest for histomorphometric analysis was limited to the secondary spongiosa. Analysis was initiated 150 μm distal to the growth plate, extending 1000 μm distally, and 50 μm from endocortical surfaces.^13^, ^46^ Images were taken at ×200, acquired by Olympus BX61 microscope (Olympus America, Inc., Center Valley, PA, USA), and analyzed using Visiopharm software (Visiopharm, Hoersholm, Denmark). Data are reported in accordance with standardized nomenclature^47^ as previously described.^13^, ^46^

### Bone marrow cultures

For each animal, femur and tibia marrow were flushed using α modified essential medium (α‐MEM; Gibco/Thermo Fisher Scientific, Waltham, MA, USA), 20% FBS (Hyclone Laboratories, Logan, UT, USA), and 1% penicillin‐streptomycin‐glutamine (PSG; 100 U/mL penicillin, 100 mg/mL streptomycin, and 2mM glutamine); marrow cells were disassociated, counted, and plated at 3 × 10^6^ cells/cm^2^ in a 60‐mm dish. Twenty‐four hours after plating, hematopoietic progenitor cells (HPCs) were isolated for osteoclast‐precursor (OCP) assays by decanting off the nonadherent cells. Fresh α‐MEM, 20% FBS (Hyclone Laboratories), and 1% PSG were added back to the bone marrow cultures, and 48 hours later adherent cells were isolated for bone marrow stromal cell (BMSC) assays. Importantly, marrow cells were not combined from animals for initial bone marrow cultures or subsequent OCP/BMSC assays; *n*‐values reported for in vitro assays represent biological replicates.

### In vitro osteoclast‐precursor assays

First passage nonadherent HPCs isolated from long bone marrow cultures were washed, and subsequently incubated with CD11b microbeads (Miltenyi Biotec, Bergisch Gladbach, Germany). An AutoMACS Sorter (Miltenyi Biotec) was employed to separate CD11b^neg^ HPCs as previously described.^9^, ^13^ CD11b^neg^ HPCs were washed, counted, and plated for assays at 1.5 × 10^5^ cells/cm^2^ in α‐MEM, 10% FBS (Hyclone Laboratories), and 1% PSG.

### TRAP stain assay

CD11b^neg^ HPCs were plated in 96‐well plates and primed for 36 hours with 10 ng/mL CSF1 (R&D Systems, Minneapolis, MN, USA), to enrich for CD11b^neg^ OCP cells having high osteoclastic potential.^48^ CD11b^neg^ OCP cultures were subsequently stimulated with fresh control (25 ng/mL CSF1; R&D Systems) or treatment (25 ng/mL CSF1 and 50 ng/mL RANKL; R&D Systems) media for 4 and 6 days; media was changed every other day. Day 4 and day 6 control cultures (25 ng/mL CSF1) and treatment cultures (25 ng/mL CSF1 and 50 ng/mL RANKL) were stained by the TRAP method as reported previously.^9^, ^13^ TRAP stain assay was carried out in triplicate (technical replicate) culture wells; four fields of view were imaged at ×100 per culture well for analysis as previously described.^9^, ^13^ The four images, taken at ×100 magnification, were methodically acquired in the same locations within the culture wells. These locations were designated at north/south/east/west and accounted for 0.166 cm^2^ of the 0.32 cm^2^ total surface area per well. TRAP+ cells with three or more nuclei were scored as osteoclasts for cytomorphometric analysis. Osteoclast cellular outcomes evaluated include number of osteoclasts enumerated within four fields of view per well (N.Oc), average osteoclast area (Oc.Ar/Oc), and nuclei number per osteoclast (N.Nc/Oc). Analysis was performed using ImageJ software, version 1.51j8, (NIH, Bethesda, MD, USA; https://imagej.nih.gov/ij/). Specific ImageJ software functions used the “Polygon selections” tool to outline cell area, the “Straight line” tool to underscore individual osteoclast cells, and the “Multi‐point” tool to denote nuclei numbers per osteoclast.

#### *Gene expression assay*

CD11b^neg^ HPCs were plated in 12‐well plates, and primed for 36 hours with 10 ng/mL CSF1 to enrich for CD11b^neg^ OCP cells having high osteoclastic potential.^48^ CD11b^neg^ OCP cultures were subsequently stimulated with fresh control (25 ng/mL CSF1; R&D Systems) or treatment (25 ng/mL CSF1 and 50 ng/mL RANKL; R&D Systems) media for 4 days. Media were changed every other day. Day 4 control cultures (25 ng/mL CSF1) and treatment cultures (25 ng/mL CSF1 and 50 ng/mL RANKL) were isolated for qRT‐PCR mRNA analysis. Gene expression assay was carried out in duplicate (technical replicate) cultures.

### In vitro bone marrow stromal cell assays

First passage BMSCs were isolated from bone marrow cultures; adherent cells were washed, trypsinized, counted, and plated for assays in α‐MEM, 10% FBS (Hyclone Laboratories), and 1% PSG. Assays were carried out in duplicate (technical replicate) cultures. Media were refreshed every other day.

#### *Cell expansion assay*

BMSCs were plated at 2.0 × 10^4^ cells/cm^2^ in 48‐well plates, and cultured in growth media (α‐MEM, 10% FBS, and 1% PSG). Cells were collected at days 2, 4, 6, 8, and 10 for cell counts.^13^, ^46^

#### *Differentiation potential assay*

BMSCs were plated at 2.0 × 10^4^ cells/cm^2^ in 12‐well plates, and cultured in growth media (α‐MEM, 10% FBS, and 1% PSG) to assess alterations in multipotent differentiation potential. Preconfluent (day 4) cultures were harvested for qRT‐PCR gene expression analysis to evaluate alterations in BMSC commitment towards the osteoblastogenic, adipogenic, and chondrogenic lineages.^13^, ^46^

#### *Osteogenesis assays*

BMSCs were plated at 1.0 × 10^5^ cells/cm^2^ in 48‐well plates, and cultured in growth media (α‐MEM, 10% FBS, and 1% PSG) for 3 days. Confluent cultures were then treated with osteogenic media (α‐MEM, 10% FBS, 1% PSG, 50 mg/mL ascorbic acid, and 10mM β‐glycerophosphate) for 5, 6, and 10 days. Mineralization was detected by the von Kossa staining method in cultures subjected to 6 and 10 days of osteogenic media treatment as previously described.^13^, ^46^ Cultures subjected to 5 days of osteogenic media treatment were harvested for qRT‐PCR analysis of *Bglap* (*Ocn*) mRNA.

### Quantitative real‐time PCR for mRNA

Femur and tibia bone marrow were flushed with TRIzol reagent (Invitrogen, Carlsbad, CA, USA), and calvaria, livers, tibias, and ileums were flash frozen, pulverized, and homogenized in TRIzol reagent. Cultures were washed twice with 1X PBS, and TRIzol reagent was directly applied. RNA was isolated by the TRIzol method following the manufacturer's instructions. Total RNA was quantified via NanoDrop 1000 (Thermo Fisher Scientific). cDNA was synthesized using TaqMan random hexamers and reverse transcription reagents (Applied Biosystems, Foster City, CA, USA) according to the manufacturer's protocol. cDNA was amplified using TaqMan gene expression primers/probes and universal PCR master mix via the StepOnePlus System (Applied Biosystems). *Gapdh* was used as an endogenous control for studies in marrow, calvaria, tibias, and ileums. *Gapdh* and *Rn18s* were used as internal controls for studies in livers. Relative quantification of data was performed via the comparative *C*_T_ method (2^−ΔΔCT^)^49^ as previously described.^9^, ^13^

### NanoString

The NanoString nCounter gene expression system (NanoString Technologies, Seattle, WA, USA) is a multiplexed probe detection system, in which a probe library is constructed with two sequence‐specific probes for each gene of interest.^50^, ^51^ mRNA expression levels are quantified via direct digital detection without the need to reverse transcribe mRNA to cDNA or the amplification of the resulting cDNA by qRT‐PCR.^50^, ^51^ The NanoString nCounter gene expression system has similar sensitivity to qRT‐PCR, superior sensitivity compared with microarrays,^50^, ^51^ and exhibits higher sensitivity for low‐abundance transcripts versus RNA‐seq.^52^ nCounter Mouse PanCancer Panel (NanoString Technologies) was applied to assess osteogenic, immune, and cytokine gene expression in long bone (femur + tibia) marrow and whole livers. Hybridization of samples was carried out, and products were run on the nCounter preparation station according to the manufacturer's instructions. Data were collected via the nCounter digital analyzer and evaluated by nSolver Analysis Software v2.6 (NanoString Technologies). Data were normalized to the geometric means of spiked‐in positive controls and built‐in housekeeping genes. Absolute quantification of mRNA was reported as normalized mRNA counts as previously described.^9^, ^13^

### qRT‐PCR for 16S rDNA

Distal ileum contents were collected at euthanasia, and bacterial DNA was isolated using the Qiagen DNeasy Powersoil Pro Kit as directed by the manufacturer (Qiagen, Hilden, Germany). qRT‐PCR reactions contained 2X Fast SYBR Green Master Mix (Applied Biosystems), 0.3 mmol/L primers, and 150 μg/mL DNA template as described previously.^9^, ^53^ qRT‐PCR protocol was executed on the StepOnePlus System (Applied Biosystems) for 30 cycles.^9^, ^53^, ^54^, ^55^

#### *SFB rDNA analysis in GF versus SFB‐monoassociated mice*

SFB was evaluated via primers: Fwd: 5′‐ GAC GCT GAG GCA TGA GAG CAT‐3′, Rev: 5′‐ GAC GGC ACG GAT TGT TAT TCA‐3′. SFB rDNA from ileum specimens of GF versus SFB‐monoassociated mice was normalized to a bacterial DNA standard (ZymoBIOMICS, Irvine, CA, USA). Twofold serial dilutions ranging from 200 μg/mL to 1 μg/mL of bacterial DNA standard were utilized in a standard curve, and relative quantification of data was carried out by the 2^−ΔCT^ method.^56^, ^57^

#### *SFB rDNA analysis in EF versus MPF mice*

The universal 16S rDNA target gene was assessed using the forward (Fwd) primer: 5′‐ ACT CCT ACG GGA GGC AGC AGT‐3′, and reverse (Rev) primer: 5′‐ ATT ACC GCG GCT GGC‐3ʼ.^53^ SFB rDNA from ileum specimens of EF versus MPF mice was normalized to the universal 16S gene, and relative quantification of SFB rDNA was performed via the comparative *C*_T_ method (2^−ΔΔCT^).^49^, ^57^

#### *Phylum‐level analyses in GF versus SFB‐monoassociated mice and EF versus MPF mice*

Primers for the phylum‐level bacterial rDNA expression were used^9^, ^53^ and are expressed as percentage abundance of the specific phylum as previously described.^9^, ^53^

### Flow cytometric analysis

Femur bone marrow, mesenteric lymph node (MLN), and liver (celiac, portal) lymph node cells were isolated, washed, and counted as previously described.^9^, ^13^

#### *Live cell analysis*

Cells were treated with FcR‐block (Miltenyi Biotec) and stained for cell surface markers. *Myeloid‐derived suppressor cells (MDSCs)*: anti‐CD11b‐APC (Miltenyi Biotec; clone REA592), anti‐Ly6G‐VioBlue (Miltenyi Biotec; clone 1A8), anti‐F4/80‐PE (Miltenyi Biotec; clone REA126), anti‐Ly6C‐FITC (Miltenyi Biotec; clone REA796). *Plasmacytoid dendritic cells (pDC)*: anti‐CD11c‐PE‐Vio770 (Miltenyi Biotec; clone REA754), anti‐MHC II‐FITC (Miltenyi Biotec; clone REA528), anti‐B220‐VioBlue (Miltenyi Biotec; clone REA755). *M1/M2 macrophages*: anti‐CD11b‐APC (Miltenyi Biotec; clone REA592), anti‐CD11c‐PE‐Vio770 (Miltenyi Biotec; clone REA754), anti‐MHC class II‐FITC (Miltenyi Biotec; clone REA528), anti‐CD64‐APC‐Vio770 (Miltenyi Biotec; clone REA286), anti‐B220‐VioBlue (Miltenyi Biotec; clone REA755), anti‐CD206‐PE (eBioscience, Santa Clara, CA, USA; clone MR6F3). Dead cells were excluded from analysis via propidium iodide viability dye (Miltenyi Biotec).

#### *CD4^+^ helper T‐cell analysis*

Cells were treated with FcR‐block (Miltenyi Biotec) and stained for cell surface markers. Cells were then treated with fixation/permeabilization buffer (eBioscience) to label intracellular transcription factors. *T*_*REG*_ *cells*: anti‐CD3‐APC‐Vio770 (Miltenyi Biotec; clone REA641), anti‐CD4‐FITC (Miltenyi Biotec; clone REA604), anti‐CD25‐PE‐Vio770 (Miltenyi Biotec; clone 7D4), and anti‐FoxP3‐PE (Miltenyi Biotec; clone REA788). *T*_*H*_ *1 cells*: anti‐CD3‐PE‐Vio770 (Miltenyi Biotec; clone REA641), anti‐CD4‐FITC (Miltenyi Biotec; clone REA604), anti‐CD183‐PE (Miltenyi Biotec; clone CXCR3‐173), anti‐T‐bet‐APC (Miltenyi Biotec; clone REA102). *T*_*H*_ *17 cells*: anti‐CD3‐APC‐Vio770 (Miltenyi Biotec; clone REA641), anti‐CD4‐FITC (Miltenyi Biotec; clone REA604), anti‐RORγT‐APC (Miltenyi Biotec; clone REA278), anti‐AHR‐PE‐Vio770 (eBioscience; clone 4MEJJ). Dead cells were excluded from analysis via e450 viability dye (Invitrogen). A minimum of 10,000 gated cells were analyzed per specimen. Data were acquired by the MACSQuant System (Miltenyi Biotec) and analyzed by FlowJo 11.0 software (TreeStar, Ashland, OR, USA).

### Serum biochemical assays

Whole blood was collected via cardiac puncture at euthanasia; serum was isolated and stored at −80°C. Carboxy‐terminal collagen crosslinks type I collagen (CTX‐1; Immunodiagnostic Systems, East Boldon, UK), osteocalcin (OCN; Alfa Aesar, Haverhill, MA, USA), C3 (Abcam, Cambridge, UK), IL17A (R&D Systems), IGF‐1 (R&D Systems), and LCN2 (R&D Systems) were assessed by ELISA following the manufacturer's protocols.

### Statistical analysis

Unpaired *t* tests were performed using GraphPad Prism 8.0 (GraphPad, La Jolla, CA, USA). Data are presented as mean ± SEM. Significance is indicated as *p < 0.050, **p < 0.010, ***p < 0.001. Power analysis consultation was carried out with the Biostatistical Unit of the Medical University of South Carolina Bioinformatics Core and was based on the authorsʼ prior experience utilizing gnotobiotic murine model systems.

## Results

### Induction of T_H_17/IL17A‐mediated immunity in SFB‐monoassociated mice

**Figure 1:**
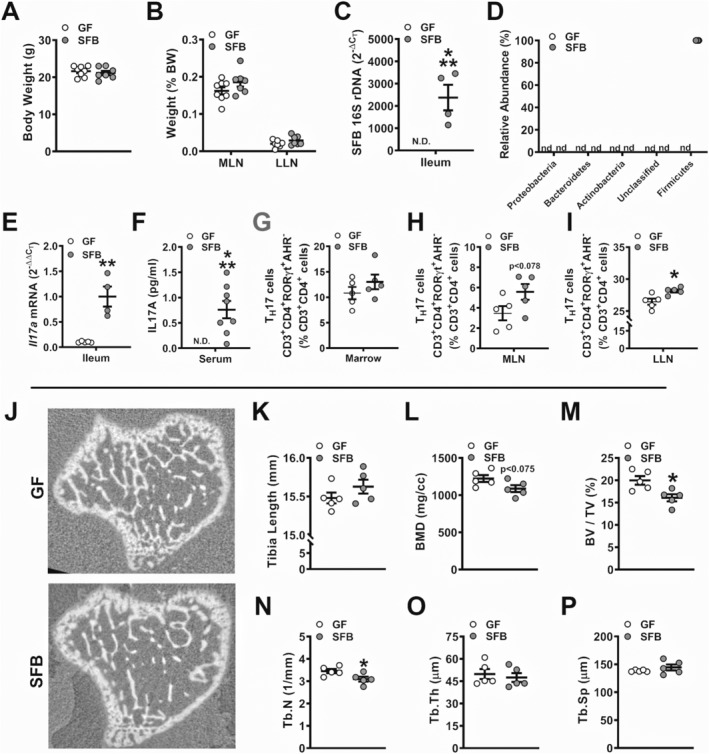
Murine phenotype and trabecular bone analysis of germ‐free versus segmented filamentous bacteria‐ (SFB‐) monoassociated mice. (*A*) Animal weight and (*B*) mesenteric lymph node (MLN) weight and liver lymph node (LLN) weight per body weight (*n* = 7 to 8/gp). (*C*) 16S rDNA gene analysis for SFB colonization in the distal ileum, reported as 2^−ΔCT,^ (*n* = 4 to 5/gp). (*D*) Phylum‐level rDNA analysis, represented as % relative abundance (*n* = 4 to 5/gp). (*E*) RNA was isolated from ileums (*n* = 4 to 5/gp) for qRT‐PCR analysis of *Il17a* mRNA. Relative quantification of mRNA was performed via the 2^−ΔΔCT^ method. (*F*) Serum was isolated from whole blood (*n* = 7 to 8/gp); ELISA analysis of IL17A levels. (*G*–*I*) Cells were isolated from (*G*) bone marrow, (*H*) MLNs, and (*I*) LLNs and stained for CD3^+^CD4^+^RORγt^+^AHR^−^ T_H_17 cells for flow cytometric analysis (*n* = 5/gp). Cell percentages are expressed relative to CD3^+^CD4^+^ lymphocyte population. (*J*–*P*) Micro‐CT analysis of proximal tibia trabecular bone (*n* = 5/gp). (*J*) Representative reconstructed cross‐sectional images, extending 50 μm distally from where analysis was initiated. (*K*) Tibia length. (*L*) BMD = trabecular bone mineral density. (*M*) BV/TV = trabecular bone volume fraction. (*N*) Tb.N = trabecular number. (*O*) Tb.Th = trabecular thickness. (*P*) Tb.Sp = trabecular separation. Unpaired *t* test; data are presented as mean ± SEM, *p < 0.050, **p < 0.010, ***p < 0.001.

There were no differences in body weight in GF versus SFB‐monoassociated mice (Fig. 1 *A*). MLN weight and liver lymph node (LLN) weight per body weight were similar in GF and SFB‐monoassociated mice (Fig. 1 *B*). 16S rDNA analysis verified SFB colonization in SFB‐monoassociated mice (Fig. 1 *C*). No detectable levels of bacterial phyla were present in SFB‐monoassociated mice, except for Firmicutes (Fig. 1 *D*). Importantly, SFB is a member of the phylum Firmicutes. SFB induction of T_H_17/IL17A‐mediated immunity was validated by elevated *Il17a* expression in the distal ileum (Fig. 1 *E*) and IL17A levels in serum (Fig. 1 *F*) of SFB‐monoassociated mice compared with GF mice. Although there were no differences in % T_H_17 cells in the marrow (Fig. 1 *G*), there was a trend towards an increase in % T_H_17 cells in the MLNs (Fig. 1 *H*), and % T_H_17 cells were significantly upregulated in the LLNs of SFB‐monoassociated versus GF mice (Fig. 1 *I*). These findings suggest that SFB may have immunomodulatory actions through both the gut and liver.

### SFB monoassociation blunts trabecular bone morphology in the postpubertal growing skeleton

Micro‐CT analysis was carried out in the tibia of GF versus SFB‐monoassociated mice to investigate SFB‐induced tissue level changes in trabecular (Fig. 1 *J*–*P*) and cortical (Supplemental Fig. S1 *A*–*C*) bone. SFB‐monoassociated mice displayed an osteopenic trabecular bone phenotype in the proximal tibia, with a trend towards decreased BMD (Fig. 1 *L*) and a 19% reduction in bone volume fraction (Fig. 1 *M*). Decreased bone volume fraction in SFB‐monoassociated mice was accredited to lower trabecular number (Fig. 1 *N*), whereas there were no alterations in trabecular thickness (Fig 1 *O*) or trabecular separation (Fig. 1 *P*). Cortical area fraction and cortical thickness were similar in the tibia mid‐diaphysis of GF versus SFB‐monoassociated mice (Supplemental Fig S1 *A*–*C*).

### SFB monoassociation enhances osteoclast differentiation and maturation potential

**Figure 2:**
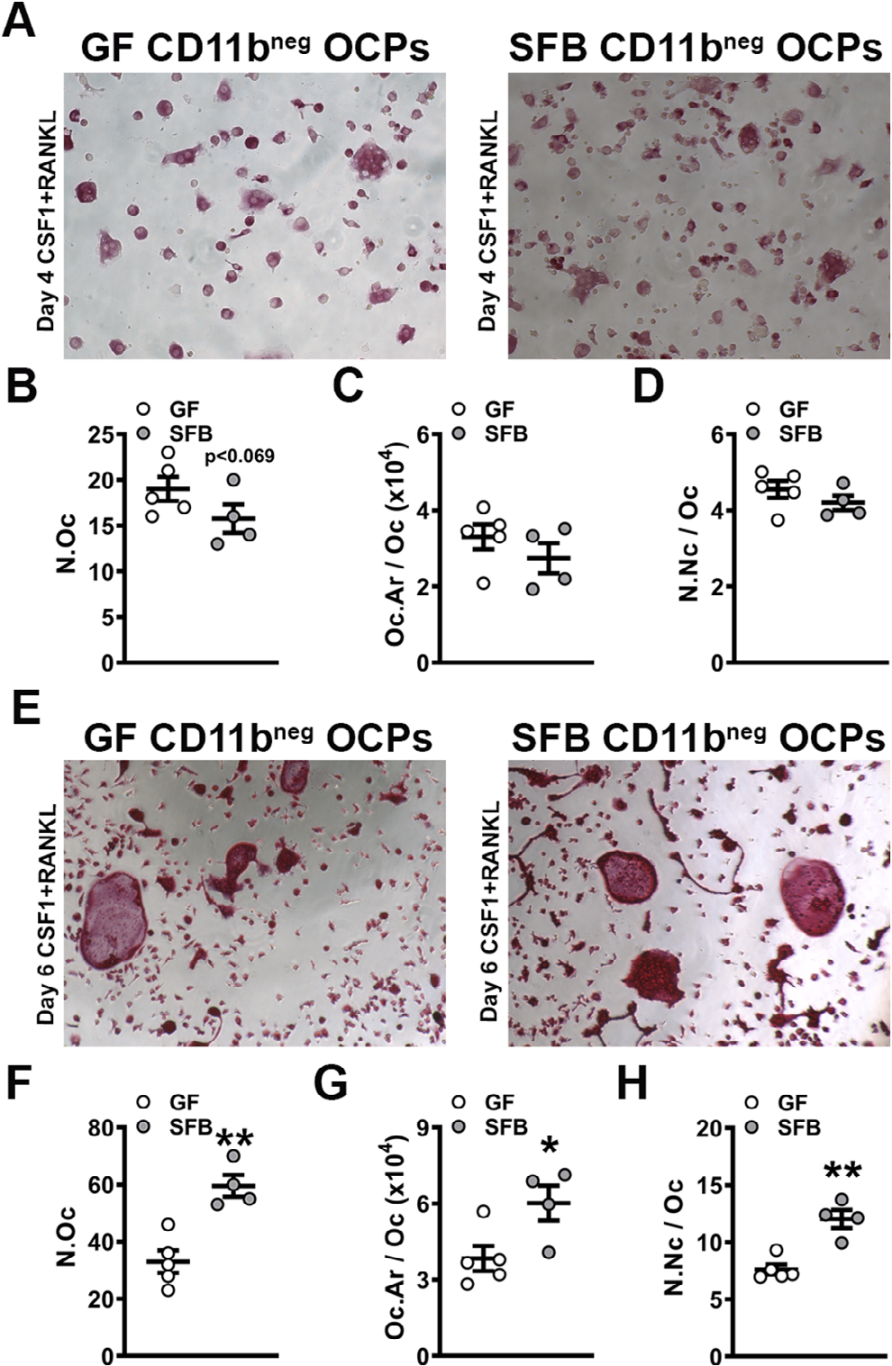
In vitro osteoclast‐precursor (OCP) cell differentiation assays in germ‐free versus segmented filamentous bacteria‐monoassociated mice. (*A*–*D*) Day 4 OCP culture tartrate‐resistant acid phosphatase stain assay (*n* = 4 to 5/gp). (*A*) Representative images (×100) of CD11b^neg^ OCP cultures stimulated with treatment (CSF1 & RANKL) media for 4 days. (*B*) N.Oc = number of osteoclasts enumerated within four fields of view per well. (*C*) Oc.Ar/Oc = average osteoclast area. (*D*) N.Nc/Oc = nuclei number per osteoclast. (*E*–*H*) Day 6 OCP culture TRAP stain assay (*n* = 4 to 5/gp). (*E*) Representative images (×100) of CD11b^neg^ OCP cultures stimulated with treatment (CSF1 & RANKL) media for 6 days. (*F*) N.Oc. (*G*) Oc.Ar/Oc. (*H*) N.Nc/Oc. Unpaired *t* test; data are presented as mean ± SEM, *p < 0.050, **p < 0.010.

Cytomorphometric analysis of day 4 OCP cell cultures from GF versus SFB‐monoassociated mice displayed no differences in osteoclast cellular endpoints (Fig. 2 *A*–*D*), suggesting that SFB immunoregulatory effects do not modulate RANKL‐stimulated early commitment of preosteoclastic cells to the osteoclast lineage. Day 6 OCP culture cytomorphometric analysis (Fig. 2 *E*–*H*) revealed alterations in OCP differentiation potential, where number of osteoclasts (N.Oc; Fig. 2 *F*), osteoclast area (Oc.Ar/Oc; Fig. 2 *G*), and number of nuclei per osteoclast (N.Nc/Oc; Fig. 2 *H*) were increased in CD11b^neg^ OCP cultures from SFB‐monoassociated versus GF mice. These data demonstrate that SFB colonization promotes a pro‐osteoclastic phenotype, which is in line with the observed impaired trabecular bone phenotype in SFB‐monoassociated versus GF mice (Fig. 1). Recognizing that IL17A is a potent pro‐osteoclastic cytokine, SFB actions supporting osteoclastogenesis may be through the induction of T_H_17/IL17A‐mediated immunity (Fig. 1).

### Validation of SFB‐induced T_H_17/IL17A‐mediated immunity in a complex commensal gut microbiota

SFB impaired the trabecular bone phenotype and enhanced the osteoclastic potential in SFB‐monoassociated versus GF mice (Fig. 1), but the impact of SFB within a complex microbiota upon the skeleton remains to be investigated. Taconic Biosciences has implemented stringent microbial guidelines for barrier locations to provide more defined SPF health models. MPF mice have the presence of SFB within a complex commensal gut microbiota, whereas EF mice have a complex commensal gut microbiota devoid of SFB colonization. Therefore, EF and MPF mice were compared to determine the influence of SFB on commensal gut microbiota osteoimmunomodulatory actions in the normal postpubertal growing skeleton.

**Figure 3:**
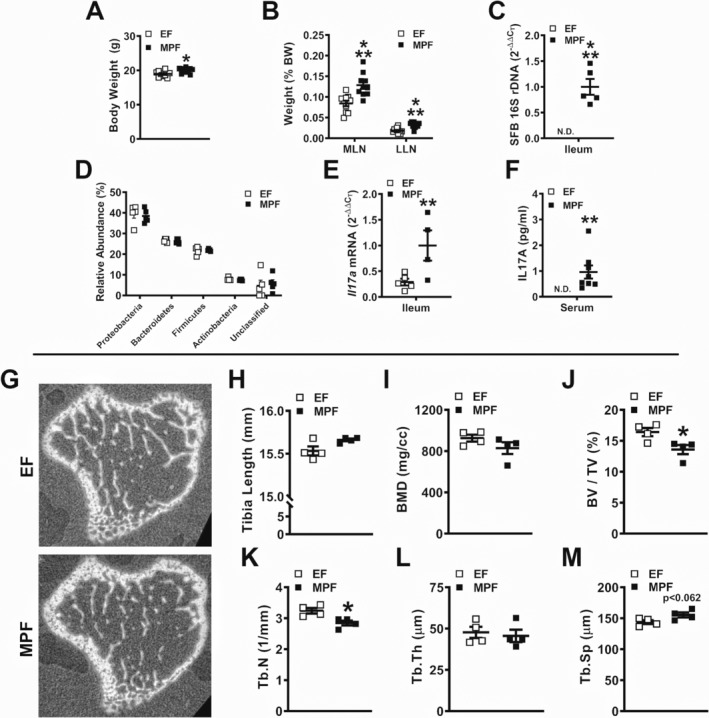
Murine phenotype and trabecular bone analysis of excluded‐flora versus murine‐pathogen‐free mice. (*A*) Animal weight and (*B*) mesenteric lymph node and liver lymph node weights per body weight (*n* = 10/gp). (*C*) 16S rDNA gene analysis for segmented filamentous bacteria colonization in the distal ileum. Relative quantification of rDNA was performed via the comparative *C*_T_ method (2^‐ΔΔCT^) (*n* = 5/gp). (*D*) Phylum‐level analysis of bacterial rDNA in the distal ileum, analysis represented as % relative abundance (*n* = 5/gp). (*E*) RNA was isolated from ileums (*n* = 5/gp) for qRT‐PCR analysis of *Il17a* mRNA. Relative quantification of mRNA was performed via 2^−ΔΔCT^. (*F*) Serum was isolated from whole blood (*n* = 7 to 8/gp); ELISA analysis of IL17A levels. (*G*–*M*) Micro‐CT analysis of proximal tibia trabecular bone (*n* = 4/gp). (*G*) Representative reconstructed cross‐sectional images, extending 50 μm distally from where analysis was initiated. (*H*) Tibia length. (*I*) BMD = trabecular bone mineral density. (*J*) BV/TV = trabecular bone volume fraction. (*K*) Tb.N = trabecular number. (*L*) Tb.Th = trabecular thickness. (*M*) Tb.Sp = trabecular separation. Unpaired *t* test; data are presented as mean ± SEM, *p < 0.050, **p < 0.010, ***p < 0.001.

Compared with EF mice, MPF mice had an increased body weight (Fig. 3 *A*). Although no differences were found in tissue weights of SFB‐monoassociated versus GF mice (Fig. 1 *B*), MLN weight and LLN weight per body weight were increased in MPF mice compared with EF mice (Fig. 3 *B*). 16S rDNA analysis confirmed SFB colonization in MPF mice, and did not detect the presence of SFB in EF mice (Fig. 3 *C*). No alterations in bacterial phyla levels were found in EF versus MPF mice (Fig. 3 *D*). Despite that SFB is a member of phylum Firmicutes, the presence of SFB did not alter the relative abundance of Firmicutes in MPF versus EF mice. These data demonstrate that the gut microbiota composition of EF and MPF mice is not dramatically different.

Elevated *Il17a* expression was detected in the distal ileum of MPF versus EF mice (Fig. 3 *E*), which is in line with prior reports demonstrating upregulated *Il17a* in the ileum of SPF mice harboring SFB.^20^, ^21^ Serum IL17A levels were increased in MPF versus EF mice (Fig. 3 *F*), further validating that SFB colonization induces T_H_17/IL17A immune response effects. Consistent with findings in the SFB‐monoassociated murine model (Fig. 1), the presence of SFB within a complex gut microbiota profoundly induces IL17A‐mediated immunity.

### SFB within a complex gut microbiota impairs trabecular bone morphology in the postpubertal growing skeleton

Micro‐CT analysis was performed in the tibia of EF and MPF mice to elucidate SFB‐induced tissue level changes in trabecular (Fig. 3 *G*–*M*) and cortical (Supplemental Fig. S2 *A*–*C*) bone. MPF mice possessed a blunted trabecular bone phenotype in the proximal tibia, characterized by a 17% reduction in bone volume fraction (Fig. 3 *J*). The inferior trabecular bone phenotype observed in MPF mice was attributed to lower trabecular number (Fig. 3 *K*) and a trend towards increased trabecular separation (Fig. 3 *M*). Cortical area fraction and cortical thickness were comparable in the tibia mid‐diaphysis of EF versus MPF mice (Supplemental Fig. S2 *A*–*C*). In line with the outcomes in the SFB‐monoassociated murine model (Fig. 1), the presence of SFB within a complex gut microbiota impairs trabecular bone growth and maturation.

### SFB within a complex gut microbiota alters innate immune response effects in lymphoid tissues draining the gut

**Figure 4:**
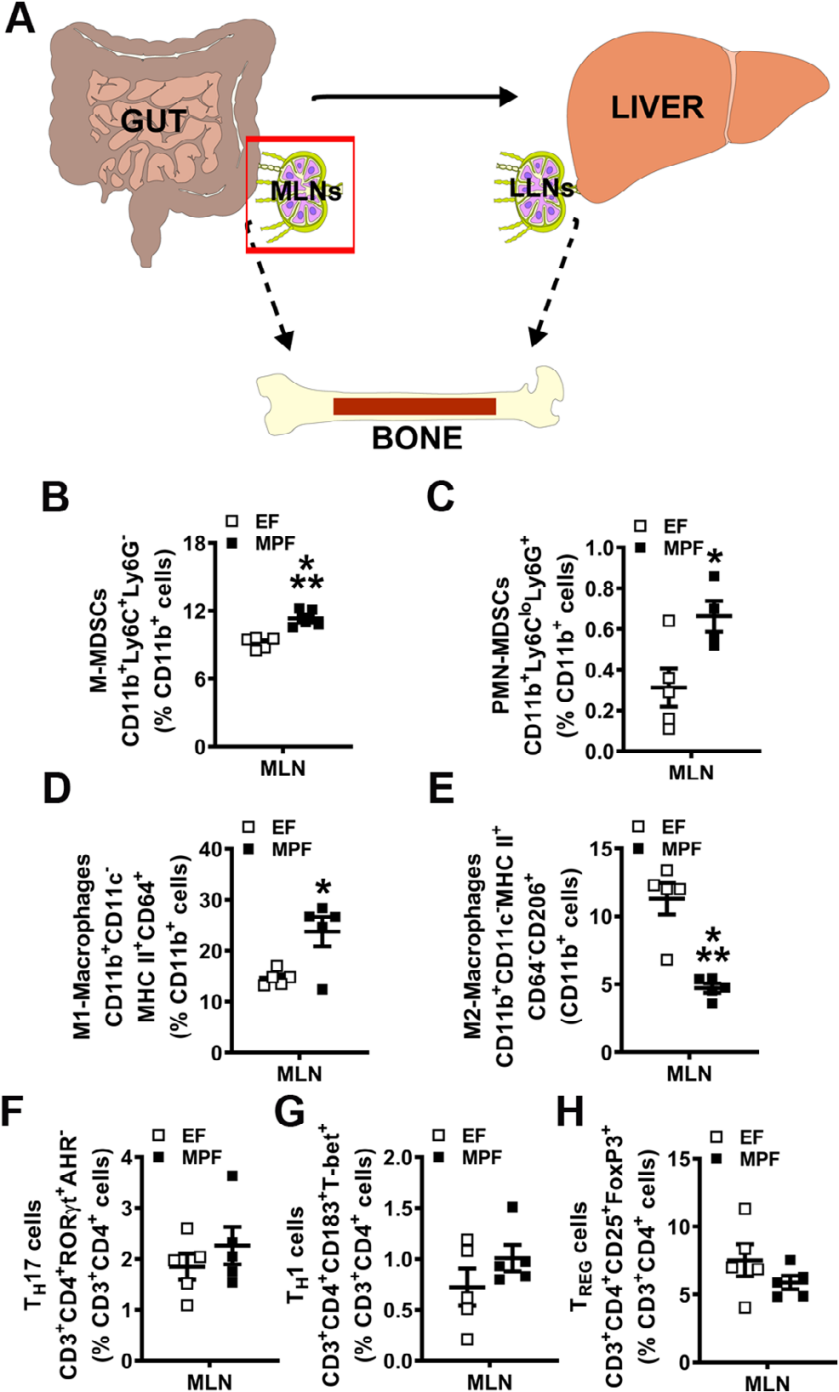
Segmented filamentous bacteria impact on immune response effects in mesenteric lymph nodes (MLNs) draining the gut. (*A*) Schematic of gut–liver–bone axis highlighting MLN cell outcomes. (*B*–*E*) MLN cells were isolated and stained for flow cytometric analysis (*n* = 5/gp) to assess the frequency of innate immune cells; cell percentages are expressed relative to CD11b^+^ monocyte population. (*B*) % CD11b^+^Ly6C^+^Ly6G^−^ monocytic myeloid‐derived suppressor cells (M‐MDSCs). (*C*) % CD11b^+^Ly6C^lo^Ly6G^+^ polymorphonuclear myeloid‐derived suppressor cells (MDSCs) (PMN‐MDSCs). (*D*) % CD11b^+^CD11c^−^MHC II^+^CD64^+^ M1‐macrophages. (*E*) % CD11b^+^CD11c^−^MHC II^+^CD64^−^CD206^+^ M2‐macrophages. (*F*–*H*) MLN cells were isolated and stained for flow cytometric analysis (*n* = 5/gp) to assess the frequency of adaptive immune cells; cell percentages are expressed relative to CD3^+^CD4^+^ lymphocyte population. (*F*) % CD3^+^CD4^+^RORγt^+^AHR^−^ T_H_17 cells. (*G*) % CD3^+^CD4^+^CD183^+^T‐bet^+^ T_H_1 cells. (*H*) % CD3^+^CD4^+^CD25^+^FoxP3^+^ T_REG_ cells. Unpaired *t* test; data are presented as mean ± SEM, *p < 0.050, ***p < 0.001. LLN = liver lymph node.

Myeloid‐lineage immune cells have been shown to be important in SFB‐induced T_H_17/IL17A mucosal immunity in the gut.^58^ However, no known studies have elucidated specific myeloid precursors that contribute to SFB immunoregulatory actions. MDSCs are immature myeloid‐cell progenitors that can give rise to dendritic cells (DCs), macrophages, and neutrophils.^59^ MDSCs originate in the bone marrow and are recruited to sites of inflammation to promote a hyperinflammatory microenvironment through several mechanisms. MDSCs are classified by two subsets, monocytic‐MDSCs (M‐MDSCs) and polymorphonuclear MDSCs (PMN‐MDSCs), which are identified by cell phenotype and immunosuppressive properties.^60^ MDSCs have been shown to regulate pathological inflammatory states such as cancer and rheumatoid arthritis.^59^, ^61^ However, the role of MDSCs in commensal gut microbiota effects on health is not well‐understood. Therefore, M‐MDSC and PMN‐MDSC subsets (Fig. 4 *B*,*C*) were evaluated by flow cytometry in the MLNs of EF and MPF mice. The % M‐MDSCs (Fig. 4 *B*) and % PMN‐MDSCs (Fig. 4 *C*) were enhanced in the MLNs of MPF mice compared with EF mice. Notably, these findings introduce MDSCs as a novel candidate regulator of SFB immunomodulatory actions in lymphoid tissues draining the gut.

Intestinal macrophages are critical for the induction of SFB‐induced T_H_17 cells and commensal antigen‐specific responses.^58^ Therefore, flow cytometry was performed to analyze M1‐ and M2‐macrophages in the MLNs of EF and MPF mice (Fig. 4 *D*,*E*). Proinflammatory M1‐macrophages were upregulated (Fig. 4 *D*), whereas anti‐inflammatory M2‐macrophages were decreased (Fig. 4 *E*) in the MLNs of MPF mice compared with EF mice. Flow cytometric analysis of CD4+ helper T‐cell subsets demonstrated no differences in frequencies of T_H_17 cells (Fig. 4 *F*), T_H_1 cells (Fig. 4 *G*), or T_REG_ cells (Fig. 4 *H*) in the MLNs of MPF versus EF mice. These findings corroborate prior studies showing that CD4^+^ effector T‐cell immunity is not altered in the MLNs of mice colonized with SFB.^28^, ^29^, ^62^

### SFB within a complex gut microbiota exacerbates proinflammatory immune responses in the liver and liver‐draining lymph nodes

**Figure 5:**
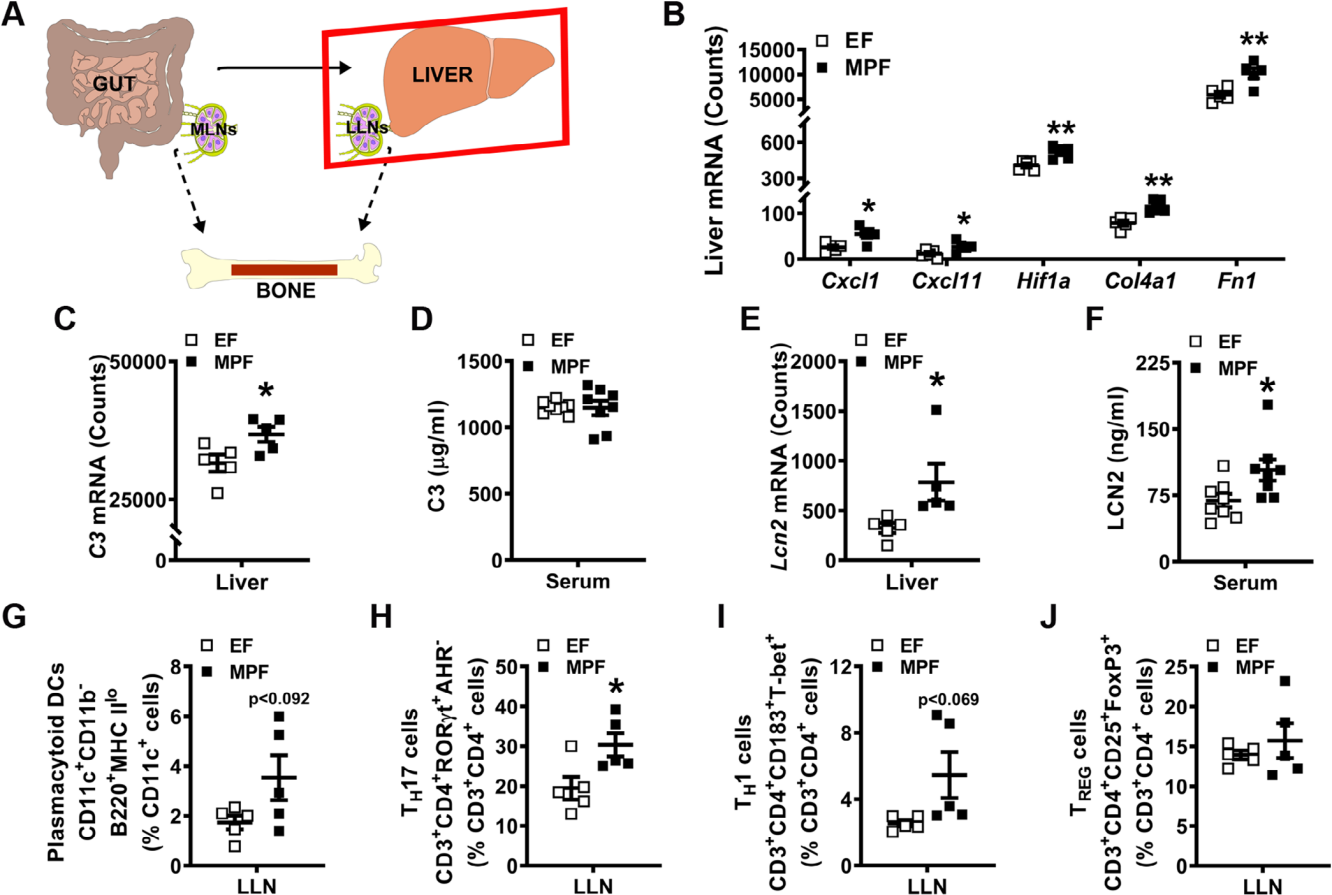
Segmented filamentous bacteria influence on liver immune response. (*A*) Schematic of gut–liver–bone axis highlighting liver outcomes. (*B*,*C*,*E*) Nanostring analysis was performed to assess mRNA counts in the liver (*n* = 5/gp). (*B*) Chemokine and profibrotic factors: *Cxcl1*, *Cxcl11*, *Hif1a*, *Col4a1*, and *Fn1* mRNA counts. (*C*) Acute‐phase reactant *C3* mRNA counts in the liver. (*D*) Serum was isolated from whole blood (*n* = 8/gp); ELISA analysis of C3 levels. (*E*) Acute‐phase reactant *Lcn2* mRNA counts in the liver. (*F*) Serum was isolated from whole blood (*n* = 8/gp); ELISA analysis of LCN2 levels. (*G*) Liver lymph node (LLN) cells were isolated and stained for flow cytometric analysis (*n* = 5/gp) to discern the frequency of CD11c^+^CD11b^−^B220^+^MHC II^lo^ plasmacytoid dendritic cells (pDCs). Cell percentages are expressed relative to CD11c^+^ monocyte population. (*H*–*J*) LLN cells were isolated and stained for flow cytometric analysis (*n* = 5/gp) to assess the frequency of adaptive immune cells. Cell percentages are expressed relative to CD3^+^CD4^+^ lymphocyte population. (*H*) % CD3^+^CD4^+^RORγt^+^AHR^−^ T_H_17 cells. (*I*) % CD3^+^CD4^+^CD183^+^T‐bet^+^ T_H_1 cells. (*J*) % CD3^+^CD4^+^CD25^+^FoxP3^+^T_REG_ cells. Unpaired *t* test; data are presented as mean ± SEM, *p < 0.050, **p < 0.010.

IL17A signaling at liver resident cells upregulates the synthesis of chemokines and profibrotic factors that contribute to proinflammatory conditions afflicting the liver.^63^, ^64^, ^65^, ^66^, ^67^, ^68^, ^69^, ^70^, ^71^ CXCL1 and CXCL11 are chemokines that have been linked to IL17A signaling effects that drive proinflammatory liver disease states.^66^, ^67^, ^68^, ^69^, ^70^, ^71^ Interestingly, CXCL11 has also been shown to endorse T_H_1 cell proinflammatory cytokine production and facilitate T_H_17 cell development in an experimental colitis model.^72^ Hypoxia inducible factor 1 (*Hif1a*), collagen type IV (*Col4a1*), and fibronectin (*Fn1*) are profibrotic factors that promote liver fibrosis.^73^, ^74^, ^75^, ^76^, ^77^, ^78^ Considering that SFB colonization within a complex gut microbiota increased serum IL17A levels (Fig. 3 *F*), gene expression studies were carried out in livers from EF and MPF mice to assess chemokines and profibrotic factors implicated in proinflammatory hepatic immune response states (Fig. 5 *B*). *Cxcl1* and *Cxcl11* were enhanced in livers of MPF mice compared with EF mice (Fig. 5 *B*). Paralleling the elevated *Cxcl1* and *Cxcl11* expression, *Hif1a*, *Col4a1*, and *Fn1* were upregulated in livers of MPF versus EF mice (Fig. 5 *B*).

Alterations in proinflammatory and profibrotic genes within the livers of MPF versus EF mice (Fig. 5 *B*) prompted investigations into acute‐phase reactants. Acute‐phase reactants are liver‐derived innate immune factors that are secreted into the circulation.^79^, ^80^, ^81^, ^82^, ^83^ Serum factors were initially classified as acute‐phase reactants based on observations during the acute‐phase of microbial infections,^84^, ^85^ but are now known to be induced during both acute and chronic inflammatory states.^85^, ^86^, ^87^ Acute‐phase reactants have antimicrobial properties that function in the elimination of pathogenic microbes, but their role in regulating commensal microbes is largely unknown. Acute‐phase reactant gene expression analysis in livers included evaluating C‐reactive protein (*Crp*), serum amyloid A 1 (*Saa1*), hepcidin antimicrobial peptide (*Hamp*), complement component 3 (*C3*), and lipocalin‐2 (*Lcn2*). Although there were no alterations in *Crp*, *Saa1*, or *Hamp* (Supplemental Fig. S3 *A*–*C*), *C3* and *Lcn2* (Fig. 5 *C*,*E*) were increased in the livers of MPF versus EF mice. Notably, these findings show that the presence of SFB within a complex gut microbiota stimulates hepatic innate immunity.

Liver‐derived acute‐phase reactants are secreted into circulation and can have endocrine signaling effects at extrahepatic sites.^82^, ^83^, ^85^, ^87^ To discern if acute‐phase reactants that were elevated in the livers of MPF versus EF mice were altered in circulation, serum was isolated from whole blood and tested for C3 and LCN2 via ELISA. No differences were detected in C3 levels (Fig. 5 *D*), but interestingly, LCN2 levels were increased in the serum of MPF versus EF mice (Fig. 5 *F*). Notably, LCN2 is an acute‐phase reactant and antimicrobial peptide that has been shown to play roles in bone development and turnover.^88^, ^89^ To corroborate that elevated LCN2 levels in the serum of MPF mice (Fig. 5 *F*) are specific to increased *Lcn2* expression in liver (Fig. 5 *E*), *Lcn2* expression was evaluated in the bone marrow, calvaria, tibia, and ileum of EF and MPF mice (Supplemental Fig. S3 *D*). There were no differences in *Lcn2* mRNA in the bone marrow, calvaria, tibia, and ileum of EF versus MPF mice (Supplemental Fig. S3 *D*), which supports the notion that elevated LCN2 levels detected in serum of MPF mice are derived from the liver. Astonishingly, these data suggest that SFB colonization upregulates acute‐phase reactants in the liver and serum, which may have endocrine signaling effects modulating bone metabolism.

Previous reports have identified LLNs, which are lymphoid tissues that drain the liver.^90^, ^91^ Of interest, liver‐derived DC migration to LLNs can profoundly elicit T‐cell activation, whereas liver‐derived DC migration to MLNs minimally affects T‐cell activation.^91^ Therefore, LLN cells were isolated from EF and MPF mice for flow cytometric analysis (Fig. 5 *G*–*J*). There was a trend towards increased % plasmacytoid DCs (pDCs) (Fig. 5 *G*) and % T_H_1 cells (Fig. 5 *I*), and % T_H_17 cells (Fig. 5 *H*) were significantly enhanced in the LLNs of MPF versus EF mice. The frequency of T_REG_ cells was similar in the LLNs of MPF versus EF mice (Fig 5 *J*). Appreciating that CXCL11 can facilitate T_H_17 cell development and support T_H_1 cell function,^72^ the upregulated *Cxcl11* detected in the liver (Fig. 5 *B*) corroborates the observed increase in % T_H_1 cells and % T_H_17 cells in the LLNs of MPF mice. The enhanced frequencies of T_H_1 and T_H_17 cells found in the LLNs of MPF versus EF mice further demonstrate that SFB colonization drives proinflammatory immune response effects in the liver, which may have implications for the postpubertal growing skeleton.

### SFB within a complex gut microbiota promotes MDSCs and T_H_17 cells in bone marrow

Investigations in GF mice and antibiotic depletion of microbiota in SPF mice have revealed that the commensal gut microbiota regulates immune cell hematopoiesis in the bone marrow environment.^10^, ^13^, ^92^ However, the role of specific microbes in commensal gut microbiota immunoregulatory effects on bone marrow hematopoiesis is currently unknown. Therefore, flow cytometric analysis was carried out to determine whether SFB modulates commensal gut microbiota actions regulating innate and adaptive immunity in the bone marrow.

**Figure 6:**
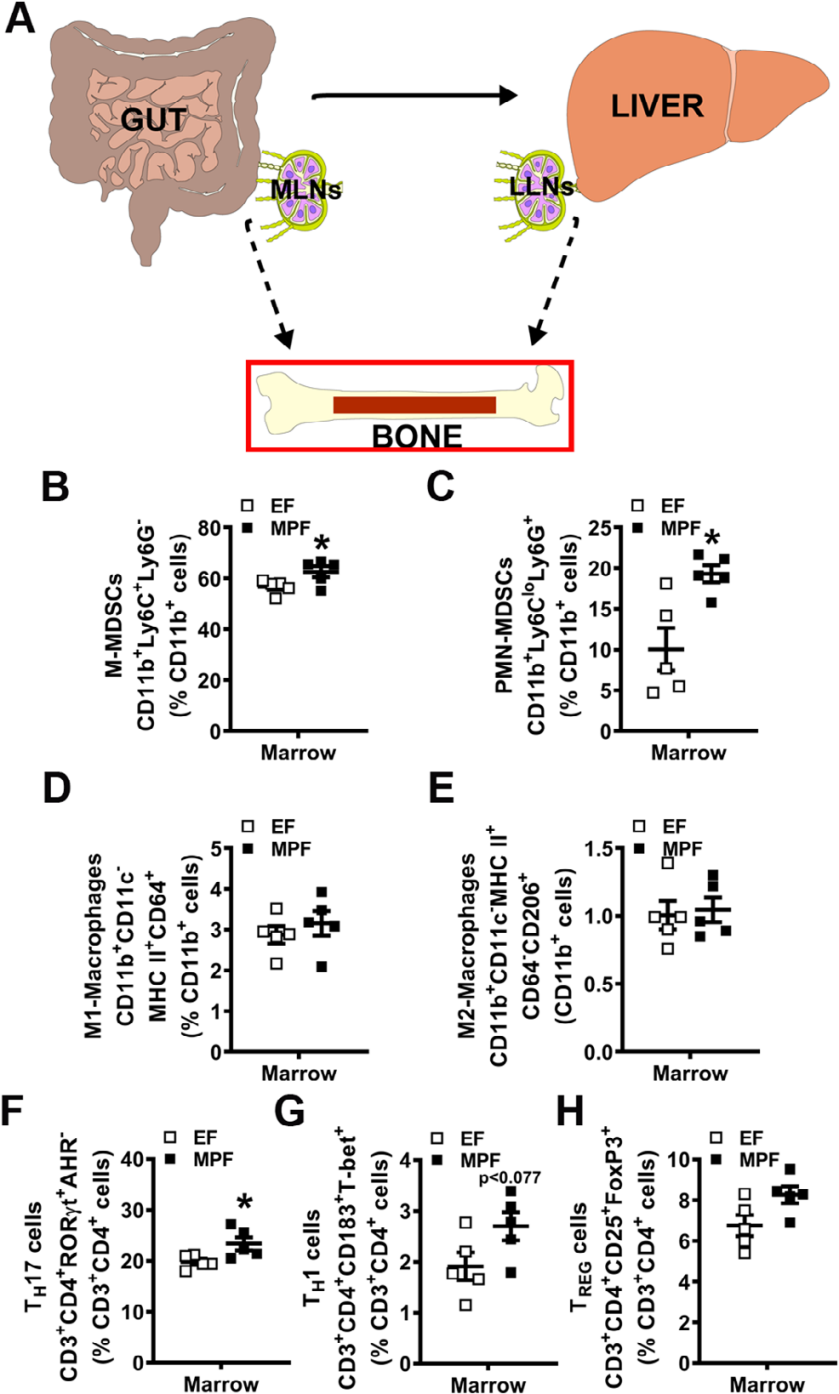
Segmented filamentous bacteria impact on immune response effects in bone marrow. (*A*) Schematic of gut–liver–bone axis highlighting bone marrow immune cell outcomes. (*B*–*E*) Bone marrow cells were isolated and stained for flow cytometric analysis (*n* = 5/gp) to assess the frequency of innate immune cells. Cell percentages are expressed relative to CD11b^+^ monocyte population. (*B*) % CD11b^+^Ly6C^+^Ly6G^−^ monocytic myeloid‐derived suppressor cells (M‐MDSCs). (*C*) % CD11b^+^Ly6C^lo^Ly6G^+^ polymorphonuclear myeloid‐derived suppressor cells (PMN‐MDSCs). (*D*) % CD11b^+^CD11c^−^MHC II^+^CD64^+^ M1‐macrophages. (*E*) % CD11b^+^CD11c^−^MHC II^+^CD64^−^CD206^+^ M2‐macrophages. (*F*–*H*) Bone marrow cells were isolated and stained for flow cytometric analysis (*n* = 5/gp) to assess the frequency of adaptive immune cells. Cell percentages are expressed relative to CD3^+^CD4^+^ lymphocyte population. (*F*) % CD3^+^CD4^+^RORγt^+^AHR^−^ T_H_17 cells. (*G*) % CD3^+^CD4^+^CD183^+^T‐bet^+^ T_H_1 cells. (*H*) % CD3^+^CD4^+^CD25^+^FoxP3^+^ T_REG_ cells. Unpaired *t* test; data are presented as mean ± SEM, *p < 0.050. MLN = mesenteric lymph node; LLN = liver lymph node.

When MDSCs are increased in the periphery under pathological states, MDSCs expand in the bone marrow and are recruited to sites of inflammation.^93^, ^94^ In line with increased MDSC subsets found in the MLNs of MPF mice (Fig. 4 *B*,*C*), the frequencies of M‐MDSCs (Fig. 6 *B*) and PMN‐MDSCs (Fig. 6 *C*) were increased in bone marrow of MPF versus EF mice. Based on findings that M1‐macrophages and M2‐macrophages were altered in the MLNs of MPF versus EF mice (Fig. 4 *D*,*E*), these cells were also analyzed by flow cytometry in the bone marrow. There were no differences in either % M1‐macrophages (Fig. 6 *D*) or % M2‐macrophages (Fig. 6 *E*) in the bone marrow of EF versus MPF mice.

Appreciating that the field of osteoimmunology has shown that specific bone marrow T‐cell subsets support osteoclastogenesis and modulate bone modeling/remodeling,^7^, ^8^ flow cytometric analysis was carried out to determine alterations in CD4^+^ T‐cell subset populations (Fig. 6 *F*–*H*). CD4^+^ helper T‐cell subsets were tested in bone marrow based on T_H_17 cells and T_H_1 cells having proinflammatory/pro‐osteoclastic effects, and T_REG_ cells having anti‐inflammatory/antiosteoclastic actions.^8^, ^95^ There was a significant upregulation in % T_H_17 cells (Fig. 6 *F*) and a trending increase of % T_H_1 cells (Fig. 6 *G*) in the marrow of MPF versus EF mice. Notably, the increased T_H_17 and T_H_1 cells found in the bone marrow (Fig. 6 *F*,*G*) paralleled the upregulated T_H_17 and T_H_1 cells detected in the LLNs (Fig. 5 *H*,*I*) of MPF versus EF mice.

### SFB within a complex gut microbiota alters osteoblastogenesis

**Figure 7:**
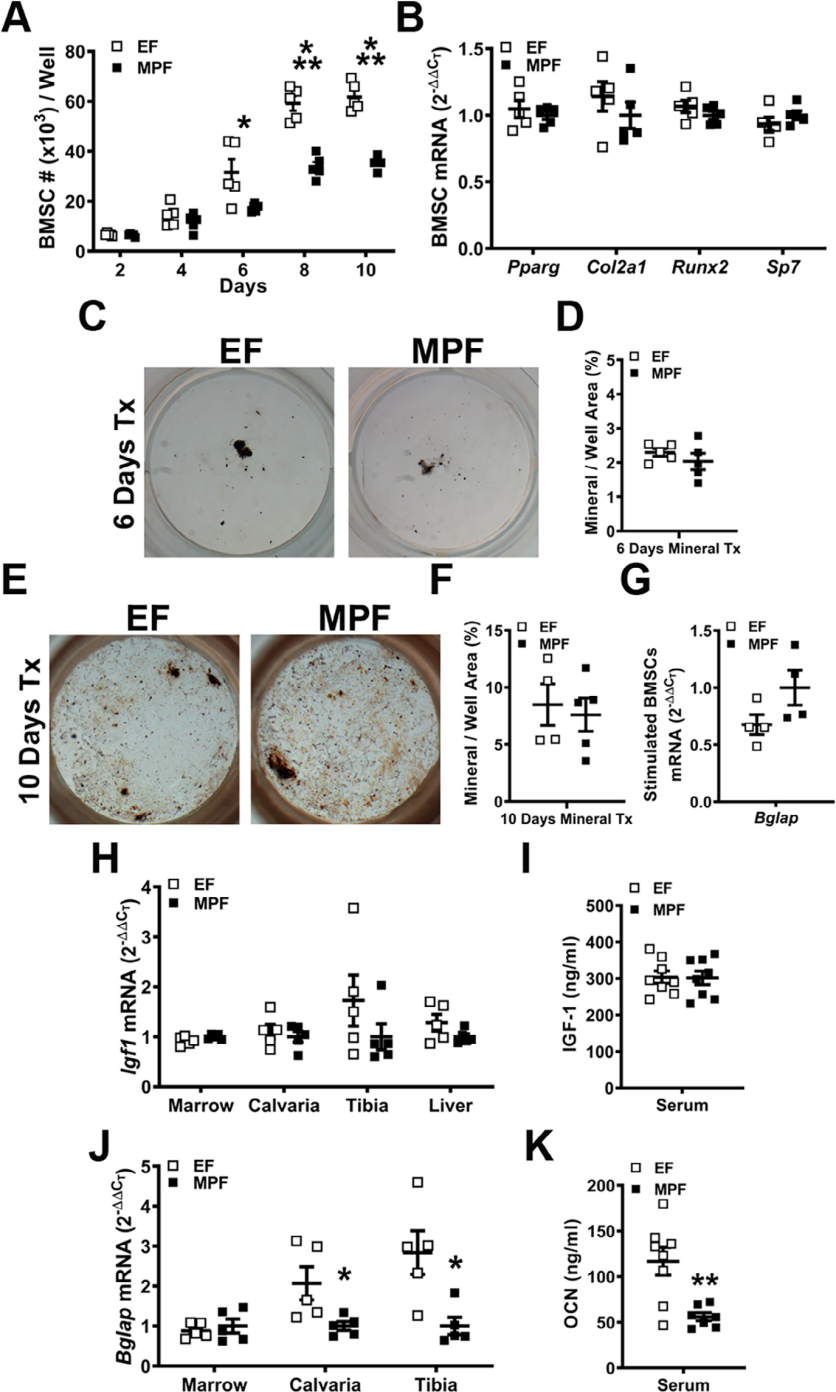
Osteoblastogenesis analyses in excluded‐flora versus murine‐pathogen‐free mice. (*A*–*G*). Bone marrow stromal cell (BMSC) in vitro osteoblastogenesis assays. Bone marrow was harvested and BMSCs isolated for in vitro assays. (*A*) BMSC expansion assay (*n* = 5/gp): cell numbers over time in culture. (*B*) BMSC differentiation potential assay (BMSCs were cultured in growth media for 4 days and harvested preconfluent for qRT‐PCR analysis) to assess multipotent differentiation potential (*n* = 5/gp). Relative quantification of mRNA was performed via the comparative CT method (2^−ΔΔCT^). (*C*–*D*) von Kossa mineralization assay (6 day osteogenic media treatment) (*n* = 5/gp). (*C*) Representative von Kossa stained culture images. (*D*) Mineralization area per well area (%). (*E*,*F*) von Kossa mineralization assay (10 day osteogenic media treatment) (*n* = 4 to 5/gp). (*E*) Representative von Kossa stained culture images. (*F*) Mineralization area per well area (%). (*G*) BMSCs stimulated with osteogenic media for 5 days were isolated for qRT‐PCR analysis of *Bglap* (*Ocn*) mRNA (*n* = 4/gp). (*H*–*K*) in vivo regulation of osteoblastogenesis. (*H*) RNA was isolated from long bone marrow, calvaria, tibia, and liver (*n* = 5/gp) for qRT‐PCR analysis of *Igf1*. (*I*) Serum was isolated from whole blood (*n* = 8/gp); ELISA analysis of IGF‐1 levels. (*J*) RNA was isolated from long bone marrow, calvaria, and tibia (*n* = 5/gp) for qRT‐PCR analysis of *Bglap*. (*K*) Serum was isolated from whole blood (*n* = 8/gp); ELISA analysis of osteocalcin levels. Unpaired *t* test; data are presented as mean ± SEM, *p < 0.050, **p < 0.010, ***p < 0.001.

Considering reports in C57BL/6 SPF versus GF mice have shown that the normal gut microbiota can blunt bone modeling/remodeling^10^, ^13^ and suppress osteoblastogenesis,^13^, ^96^ alterations in osteoblastogenesis were evaluated in EF versus MPF mice (Fig. 7). BMSCs, multipotent mesenchymal‐progenitor cells residing in the marrow, were isolated to interpret SFB‐induced changes in osteoblastic potential (Fig. 7 *A*–*G*). BMSC expansion over time was suppressed in MPF versus EF cultures (Fig. 7 *A*). To assess alterations in multipotent differentiation potential, BMSCs were cultured in growth media for 4 days and harvested preconfluent for gene expression analysis (Fig. 7 *B*). Preconfluent day 4 BMSC cultures from MPF versus EF mice showed no differences in the commitment to the adipogenic (*Pparg*), chondrogenic (*Col2a1*), or osteogenic (*Runx2*, *Sp7*) lineages (Fig. 7B). To evaluate alterations in osteoblast differentiation and function, confluent BMSC cultures from EF and MPF mice were stimulated with osteogenic media for von Kossa mineralization assays (Fig. 7 *C*–*F*). Mineralization outcomes were similar in MPF versus EF BMSC cultures subjected to 6 days (Fig. 7 *C*,*D*) and 10 days (Fig. 7 *E*,*F*) of osteogenic media treatment. As a means to validate the lack of differences in the osteoblastic potential discerned through the von Kossa assays (Fig. 7 *C*–*F*), confluent BMSC cultures from EF and MPF mice were subjected to 5 days osteogenic media treatment for qRT‐PCR analysis of *Bglap* (*Ocn*) mRNA (Fig. 7G). MPF versus EF BMSC cultures demonstrated no difference in the expression of *Bglap* (*Ocn*), which is a surrogate marker for mature osteoblast function.

To investigate SFBʼs effects on in vivo osteoblastogenesis, gene expression studies were carried out for osteogenic signaling factors (*Igf1*, *Fgf2*) and markers for mature osteoblast function [*Bglap* (*Ocn*)] in bone marrow, calvaria, and tibia (Fig. 7, Supplemental Fig. S4). Although these bone tissues are heterogeneous in cellular makeup, calvaria and tibia have a more homogenous stromal‐osteoblastic cellular composition that may better reflect osteoblastic lineage gene expression. There were no alterations in *Igf1* mRNA expression in bone marrow, calvaria, and tibia from EF versus MPF mice (Fig. 7 *H*). Recognizing that prior reports have shown that the commensal gut microbiota modulates the IGF‐1 axis,^11^, ^12^, ^13^ liver *Igf1* mRNA and serum IGF‐1 levels were evaluated. Corroborating the lack of alterations in *Igf1* expression in marrow, calvaria, and tibia, liver *Igf1* mRNA (Fig. 7 *H*) and serum IGF‐1 (Fig. 7 *I*) outcomes were not different in EF versus MPF mice. Similarly, *Fgf2* expression was not altered in bone marrow, calvaria, and tibia of EF versus MPF mice (Supplemental Fig. S4 *A*). Although there were no differences in the osteogenic factors, *Igf1* and *Fgf2*, *Bglap (Ocn)* expression was downregulated in calvaria and tibia of MPF mice (Fig. 7 *J*). Paralleling the decreased *Bglap* (*Ocn*) expression found in calvaria and tibia, serum OCN levels were reduced in MPF versus EF mice (Fig. 7 *K*). These in vivo study findings show that SFB has antiosteogenic effects on commensal gut microbiota osteoimmunomodulatory actions. Considering the lack of differences found in osteoblastic potential through the in vitro BMSC culture system, the decreased in vivo *Bglap*/OCN outcomes in MPF mice suggest that SFB antiosteoblastic effects rely on the in vivo environment. It appears that the presence of SFB in a complex gut microbiota alters circulating factors and/or local signaling molecules in the bone marrow, which suppress osteogenesis in the in vivo environment.

### SFB within a complex gut microbiota enhances osteoclast potential

**Figure 8:**
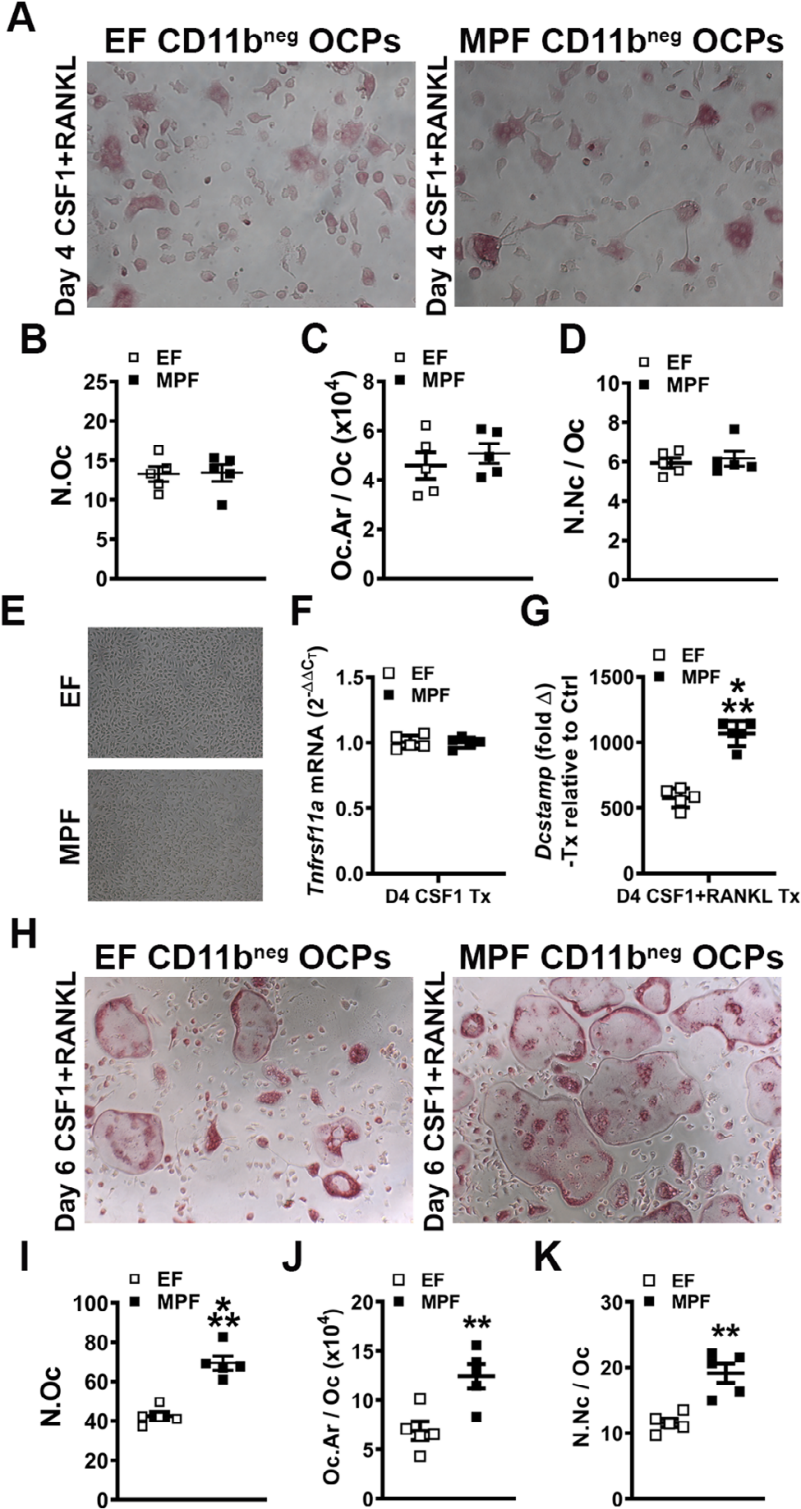
In vitro osteoclast‐precursor (OCP) cell differentiation assays in EF versus MPF mice. (*A*–*D*) Day 4 OCP culture tartrate‐resistant acid phosphatase stain assay (*n* = 5/gp). (*A*) Representative images (×100) of CD11b^neg^ OCP cultures stimulated with treatment (CSF1 & RANKL) media for 4 days. (*B*) N.Oc = number of osteoclasts enumerated within four fields of view per well. (*C*) Oc.Ar/Oc = average osteoclast area. (*D*) N.Nc/Oc = nuclei number per osteoclast. (*E*) Representative images (×100) of CD11b^neg^ OCP cultures stimulated with control (CSF1) media for 4 days. (*F*–*G*) qRT‐PCR gene expression studies were carried out in CD11b^neg^ OCP cultures at day 4 to detect early transcription level alterations in RANKL‐stimulated osteoclast differentiation (*n* = 5/gp). (*F*) *Tnfrsf11a (Rank)* mRNA. Relative quantification of mRNA was performed via the comparative CT method (2^−ΔΔCT^). (*G*) *Dcstamp* mRNA. Relative quantification of mRNA was performed via 2^−ΔΔCT^; data expressed as treatment (CSF1 and RANKL) fold change relative to control (CSF1). (*H*–*K*) Day 6 OCP culture TRAP stain assay (*n* = 5/gp). (*H*) Representative images (×100) of CD11b^neg^ OCP cultures stimulated with treatment (CSF1 & RANKL) media for 6 days. (*I*) N.Oc. (*J*) Oc.Ar/Oc. (*K*) N.Nc/Oc. Unpaired *t* test; data are presented as mean ± SEM, **p < 0.010, ***p < 0.001.

Paralleling the GF versus SFB‐monoassociated CD11b^neg^ OCP culture outcomes at day 4, cytomorphometric analysis of TRAP stained day 4 CD11b^neg^ OCP cultures from EF versus MPF mice displayed no alterations in osteoclast cellular endpoints (Fig. 8 *A*–*D*). Day 4 OCP culture gene expression analysis was performed to evaluate differences in critical osteoclastic genes. CSF1‐stimulated control cultures (Fig. 8 *E*) were tested for *Tnfrsf11a* (*Rank*) (Fig. 8 *F*), the RANKL receptor, and a surrogate marker for preosteoclast/osteoclast cells in vitro. CSF1 signaling at its cognate receptor induces the expression of RANK,^97^ allowing for the expansion and differentiation of osteoclast precursor cells.^98^ *Tnfrsf11a* expression was similar in EF and MPF cultures, suggesting that SFB does not alter osteoclast precursor cell expansion/differentiation.

Treatment over control analysis was performed in RANKL+CSF1 treatment cultures relative to CSF1 control cultures to evaluate *Dcstamp*, a RANKL‐induced transmembrane protein important for osteoclast fusion. RANKL treatment more profoundly upregulated *Dcstamp* in CD11b^neg^ OCP cultures from MPF versus EF mice (Fig. 8 *G*). This suggests that the SFB immunostimulation promotes RANKL‐induced osteoclast fusion. Validating gene‐level alterations in *Dcstamp* (Fig. 8 *G*), day 6 OCP TRAP^+^ cytomorphometric analysis (Fig. 8 *H*–*K*) displayed an increased number of osteoclasts (Fig. 8 *I*), osteoclast area (Fig. 8 *J*), and number of nuclei per osteoclast (Fig. 8 *K*) in cultures from MPF versus EF mice. These data support the notion that SFB colonization enhances the commensal gut microbiota actions promoting osteoclastogenesis.

**Figure 9:**
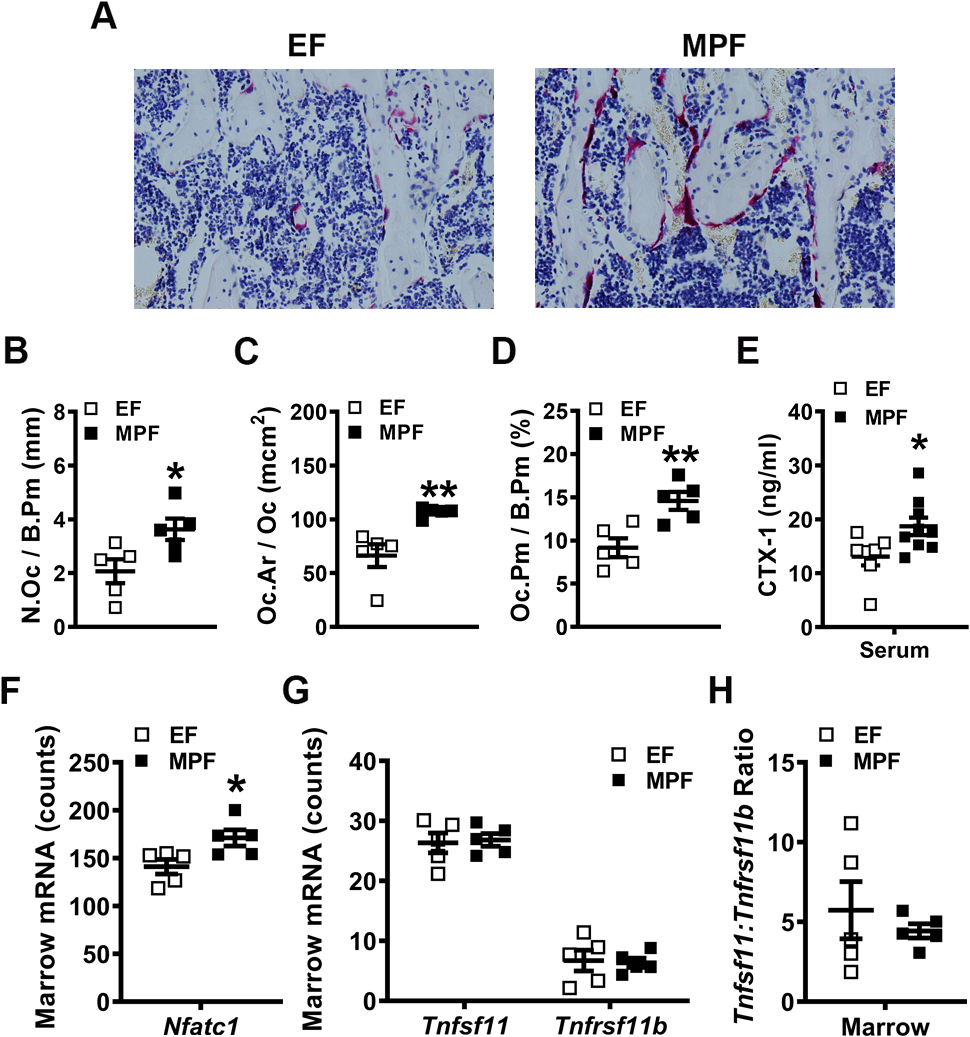
In vivo osteoclastogenesis investigations in excluded‐flora versus murine‐pathogen‐free mice. (*A*–*D*) Histomorphometric analyses of tartrate‐resistant acid phosphatase+ (TRAP+) osteoclast cellular endpoints in the proximal tibia trabecular bone secondary spongiosa (*n* = 5/gp). (*A*) Representative images of TRAP‐stained secondary spongiosa in proximal tibia (×400). (*B*) N.Oc/B.Pm = osteoclast number per bone perimeter. (*C*) Oc.Ar/Oc = average osteoclast size. (*D*) Oc.Pm/B.Pm = osteoclast perimeter per bone perimeter. (*E*) Serum was isolated from whole blood (*n* = 7 to 8/gp); ELISA analysis of CTX‐1 levels. (*F*–*H*) Long bone marrow was harvested for gene expression analysis (*n* = 5/gp). (*F*) *Nfatc1* mRNA counts. (*G*) *Tnfsf11*(*Rankl*) and *Tnfrsf11b*(*Opg*) mRNA counts. (*H*) *Tnfsf11*(*Rankl*):*Tnfrsf11b*(*Opg*) ratio. Unpaired *t* test; data are presented as mean ± SEM, *p < 0.050, **p < 0.010.

To determine if osteoclastogenesis is elevated with the presence of SFB in vivo, histomorphometric analysis of TRAP‐stained proximal tibia sections was performed (Fig. 9 *A*–*D*). There was an increased number of osteoclasts lining the trabecular bone perimeter (N.Oc/B.Pm) in MPF versus EF mice (Fig. 9 *B*), which suggests that SFB enhances the commitment of monocyte/myeloid cells to the osteoclast lineage. Osteoclast size (Oc.Ar/Oc) was larger in MPF versus EF mice (Fig. 9 *C*), which indicates that SFB supports osteoclast maturation. The increased N.Oc/B.Pm and Oc.Ar/Oc both contributed to a greater osteoclast perimeter per bone perimeter (Oc.Pm/B.Pm; Fig. 9 *D*) in MPF mice. The increased N.Oc/B.Pm (Fig. 9 *B*) and Oc.Ar/Oc (Fig. 9 *C*) findings in the proximal tibia of MPF versus EF mice importantly corroborate the enhanced osteoclast numbers (Fig. 8 *I*) and maturation potential (Fig. 8 *J*–*K*) found in the day 6 OCP culture system. Serum CTX‐1 levels were assessed via ELISA to address osteoclast function. CTX‐1 levels in serum were significantly elevated in MPF versus EF mice (Fig. 9 *E*), demonstrating that SFB within a complex commensal gut microbiota enhances osteoclast resorptive function.

Gene expression studies were executed in bone marrow isolates to further characterize SFB pro‐osteoclastic actions in vivo. *Nfatc1*, a transcription factor critical for RANKL‐induced osteoclastogenesis, was enhanced in marrow of MPF versus EF mice (Fig. 9 *F*). Importantly, the *Nfatc1* outcomes were consistent with the observed increased in vivo osteoclast differentiation/maturation (Fig. 9 *A*–*D*) and function (Fig. 9 *E*) in MPF mice. The *Tnfsf11* (*Rankl*)/*Tnfrsf11b* (*Opg*) axis was also evaluated (Fig. 9 *G*,*H*) to determine whether alterations in critical and necessary osteoclastic signaling factors mediate the pro‐osteoclastic phenotype found in MPF versus EF mice. The expression of *Tnfsf11* (*Rankl*) and *Tnfrsf11b* (*Opg*) (Fig. 9 *G*) was similar, and there were no differences in the *Tnfsf11* (*Rankl*)/*Tnfrsf11b* (*Opg*) ratio (Fig. 9 *H*) in the marrow of EF versus MPF mice.

## Discussion

**Figure 10:**
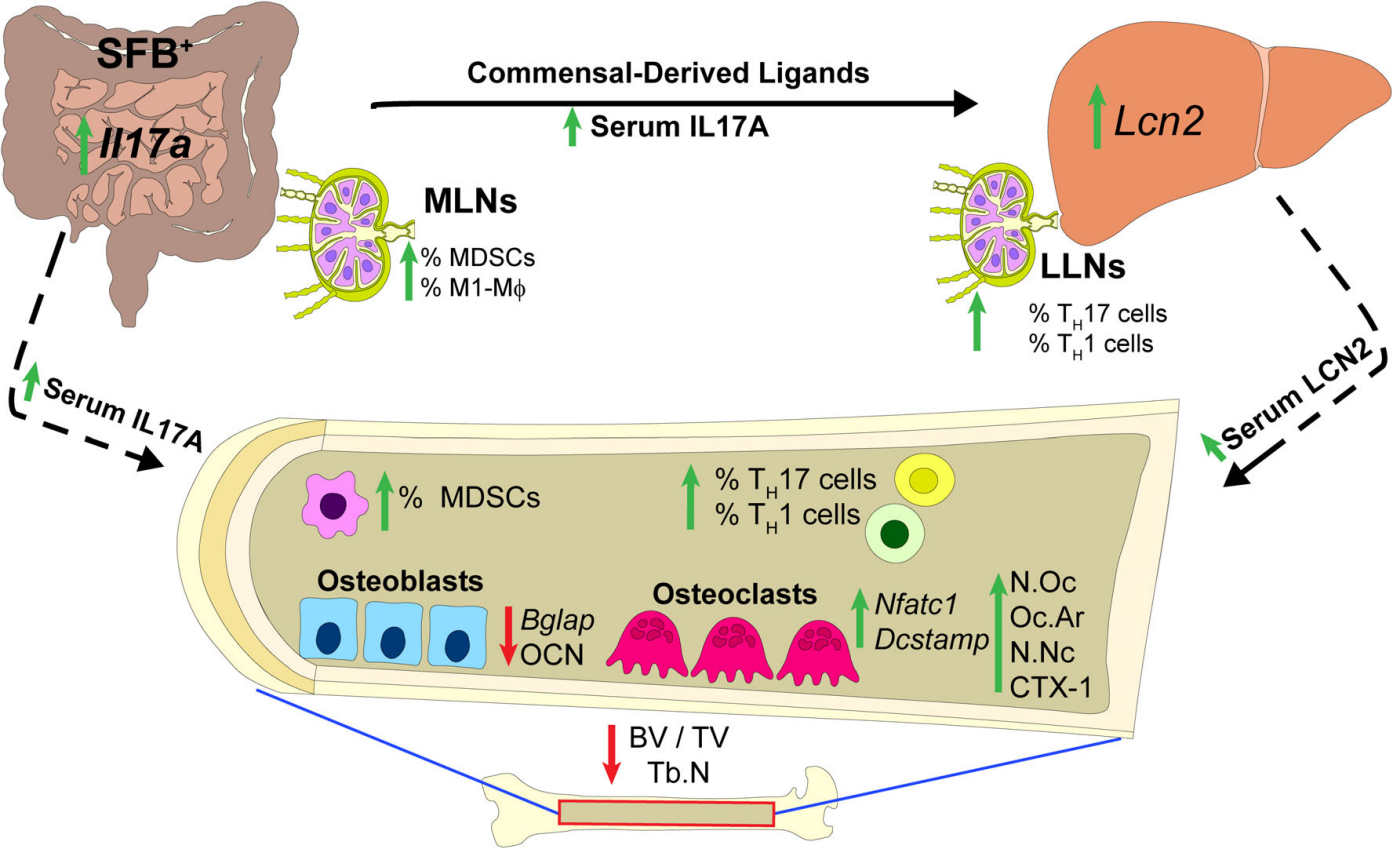
Schematic of segmented filamentous bacteria (SFB) osteoimmunoregulatory effects in postpubertal skeletal development. The presence of SFB drives the expression of *Il17a* in the ileum. SFB colonization in the gut promotes commensal‐derived molecular ligands/metabolites and circulating IL17A, supporting innate immune responses in the mesenteric lymph nodes (MLNs) and in the liver. SFB upregulated LCN2 in the liver and serum, which supports the notion that SFB osteoimmunomodulatory actions are mediated in part through a gut–liver–bone axis. SFB increased proinflammatory immune response effects in the lymphoid tissues draining the gut and liver, as well as the bone marrow, which suppressed osteoblastogenesis, enhanced osteoclastogenesis, and blunted trabecular bone microarchitecture. SFB critically impacts commensal gut microbiota immunomodulatory actions regulating normal postpubertal skeletal growth and maturation. BV/TV = trabecular bone volume fraction; LLN = liver lymph node; MDSCs = myeloid‐derived suppressor cells; N.Nc = number of nuclei; N.Oc = number of osteoclasts enumerated within four fields of view per well; Oc.Ar = osteoclast area; OCN = osteocalcin; Tb.N = trabecular number.

The current study elucidates that SFB colonization critically alters commensal gut microbiota immunomodulatory actions, which impair trabecular bone microarchitecture in the postpubertal growing skeleton. Although prior reports have discerned that the normal gut microbiota regulates physiological bone metabolism in the healthy skeleton,^10^, ^11^, ^12^, ^13^ this is the first known study to delineate that specific commensal microbes critically influence commensal gut microbiota osteoimmunoregulatory effects (Fig. 10). SFB colonization upregulated *Il17a* expression in the distal ileum, enhanced serum IL17A levels, and increased innate immune responses (M‐MDSCs, PMN‐MDSCs, M1‐macrophages) within the MLNs draining the gut. SFB presence in the commensal gut microbiota promoted acute‐phase reactant *Lcn2* expression within the liver and elevated serum LCN2 levels. Recognizing that LCN2 is antimicrobial peptide synthesized in the liver, this implies that SFB colonization exacerbates commensal gut microbiota‐derived ligands and circulating IL17A, which pass through the portal venous circulation to stimulate the hepatic innate immune response. Enhanced hepatic chemokine and profibrotic factor expression with the presence of SFB supported an elevated adaptive immune response (T_H_17, T_H_1 cells) within lymph nodes draining the liver. SFB‐induction of *Il17a* in the gut and *Lcn2* in the liver led to increased circulating levels of IL17A and LCN2. Considering that IL17A and LCN2 have pro‐osteoclastic/antiosteoblastic actions, SFB actions blunting trabecular bone development appear to be mediated through direct effects in the gut and indirect immune response effects in the liver (Fig. 10). This research reveals that specific microbes critically impact commensal gut microbiota immunomodulatory actions regulating normal postpubertal skeletal growth and maturation.

The current report is the first known study to utilize a monoassociated animal model to discern osteoimmunoregulatory effects of a single commensal gut microbe on the healthy, growing skeleton. SFB monoassociation in GF mice was strategically carried out in 5‐week‐old weanling mice, because studies have demonstrated that SPF mice are spontaneously colonized by SFB several days following weaning.^21^, ^25^, ^26^, ^27^ Female C57BL/6 mice typically reach puberty by 5 weeks of age, which is considered the murine onset of postpubertal skeletal development.^36^, ^37^, ^38^ SFB colonization at age 5 weeks and subsequent euthanization of the mice at age 9 weeks, facilitated evaluating the impact of SFB on postpubertal skeletal growth and maturation. Importantly, the postpubertal phase is a developmental period associated with robust bone modeling that accounts for approximately 40% of peak bone mass.^39^, ^40^, ^41^, ^42^ In addition to interest in the postpubertal growth of the skeleton, mice were sacrificed at age 9 weeks, because it has been reported that SFB colonization is transient during development. SFB colonization peaks at around 60 days^21^ and declines significantly at age 12 to 16 weeks in mice.^21^, ^27^ Taconic Biosciencesʼ application of stricter microbial guidelines for SPF barrier facilities provided for the unique opportunity to study C57BL/6 T SPF mice with and without SFB colonization. Therefore, the current study was able to discern the impact of SFB within a complex commensal gut microbiota on osteoimmune processes in the postpubertal developing skeleton. Although a prior report applied different Taconic Biosciences’ SPF models to show that SFB exacerbates commensal gut microbiota actions in autoimmune arthritis,^35^ our investigation found that the presence of SFB promotes pro‐osteoclastic/antiosteoblastic effects in the healthy, growing skeleton. Further studies are needed to discern whether SFB‐derived ligands or SFB‐induced changes in commensal gut microbiota composition are responsible for osteoimmunoregulatory effects impairing normal skeletal growth and maturation.

The current report is also the first known study to begin to delineate the role of MDSCs in SFB immunomodulatory effects. Elevated MDSCs in the bone marrow of MPF mice supports the notion that MDSC subsets are expanding in the bone marrow to be recruited to gut‐draining MLNs when SFB is present in a complex gut microbiota. MDSCs derive from the bone marrow where they make‐up roughly 30% of the normal marrow composition.^59^, ^99^ MDSCs produce proinflammatory cytokines that can induce naïve CD4^+^ cells into T_H_17/IL17A^+^ cells.^100^ Moreover, MDSCs are profoundly increased in proinflammatory states and have been shown to act as osteoclast precursors under pathologic conditions.^101^, ^102^, ^103^ SFB presence in the complex commensal gut microbiota promoted MDSC expansion in bone marrow, which may have contributed to the increased marrow T_H_17 cells and enhanced osteoclastic phenotype found in MPF versus EF mice.

It has been well‐established that SFB induces T_H_17/IL17A‐mediated immunity in the lamina propria of the small intestine.^21^, ^28^, ^29^, ^30^ SFB has also been shown to play a part in regulating macrophages,^58^, ^104^ DCs,^29^, ^105^, ^106^ and effector T_H_1 polarization.^21^, ^35^, ^104^, ^107^ Specifically, SFB utilizes intestinal phagocytes such as macrophages and DCs that are vital for commensal antigen‐specific responses and T_H_17 induction.^58^, ^105^ Although secondary lymphoid tissues have been considered expendable in SFB‐induced T_H_17 cell maturation,^29^, ^62^ these tissues are still required to promote T_H_17 specificity to SFB‐derived antigens.^28^ The upregulated proinflammatory M1‐macrophages and decreased anti‐inflammatory M2‐macrophages found in the MLNs of MPF versus EF mice support the outcomes of prior reports, which discerned that macrophages play a pivotal role in SFB‐induced host immunity.

T_H_17/IL17A‐mediated immunity has been associated with promoting liver inflammation and fibrosis.^63^, ^64^, ^65^, ^66^, ^67^, ^68^, ^69^, ^70^, ^71^ IL17A signaling at liver resident cells can enhance proinflammatory chemokine release, including CXCL1 and CXCL11,^66^, ^67^, ^68^, ^69^, ^70^, ^71^ and promote profibrotic effects.^63^, ^64^, ^66^, ^67^, ^71^ Moreover, SFB‐induced IL17A signaling has been shown to exacerbate obesity‐induced liver damage in nonalcoholic fatty liver disease in vivo.^108^ *Cxcl1*, *Cxcl11*, *Hif1a*, *Col4a1*, and *Fn1* were unexpectedly elevated in the liver of MPF versus EF mice, suggesting that SFB within the complex commensal gut microbiota leads to proinflammatory innate immune response effects in the liver. Astonishingly, these findings are in line with seminal reports demonstrating that CXCL1, CXCL11, HIF1A, COL4A1, and FN1 are implicated in liver fibrosis.^66^, ^73^, ^74^, ^75^, ^76^, ^77^, ^78^, ^109^, ^110^, ^111^

The liver is exposed to commensal gut microbiota‐derived ligands/metabolites through the portal venous circulation.^112^, ^113^, ^114^ DCs are critical in presenting antigens to promote T‐cell activation in liver‐draining lymph nodes.^91^ Our findings of elevated pDCs, T_H_17 cells, and T_H_1 cells in the LLNs of MPF mice suggest that SFB colonization drives hepatic innate immune responses leading to increased proinflammatory T_H_17 cells and T_H_1 cells in liver‐draining lymph nodes. With upregulated T_H_17 cells detected in the LLNs and bone marrow of MPF mice, it is unclear whether these cells are SFB‐specific T_H_17 cells or whether the local microenvironment promotes non‐antigen‐specific T_H_17 cell induction. Although seminal studies have found that the MLNs are critical and necessary for oral antigen/food tolerance through the adaptive immune response,^115^, ^116^ this concept may also hold true in the liver and its draining lymph nodes to have tolerance towards commensal‐derived ligands/antigens. Commensal gut microbiota‐derived ligands, filtered through the liver, may stimulate hepatic innate immune cells to respond and migrate to the LLNs to promote an adaptive immune response that is vital in the mutualistic relationship between microbiota and host. The current finding of SFB colonization driving such actions within the liver and LLNs demonstrates that the liver may be a critical immune organ for SFB‐induced immunomodulatory actions impacting the postpubertal growing skeleton.

Gut microbiota‐derived ligands/metabolites are known to directly modulate inflammatory responses in liver‐residing cells, such as the synthesis of proinflammatory cytokines and acute‐phase reactants.^79^, ^80^, ^81^, ^82^, ^83^ LCN2, an iron‐sequestering antimicrobial peptide, is synthesized by the liver as an acute‐phase reactant.^117^, ^118^, ^119^, ^120^, ^121^ Microbe ligand recognition and inflammation upregulate the synthesis of LCN2.^118^, ^119^, ^120^, ^122^ Central to the current investigation, findings that LCN2 is upregulated in the liver and serum, but not in the gut and skeletal tissues of MPF versus EF mice, demonstrate a novel immunoregulatory mechanism driven by SFB within the commensal gut microbiota. Resident liver cells secrete a quarter of the circulating LCN2 under basal conditions^118^ and are responsible for 90% of circulating LCN2 postbacterial infection.^117^, ^118^ Although acute‐phase reactants have antimicrobial properties that function in the elimination of pathogenic microbes, their role in regulating commensal microbes is unclear. Notably, our findings introduce LCN2 as a candidate immunoregulator of SFB colonization, highlighting the role of gut–liver crosstalk in maintaining a homeostatic relationship with the commensal gut microbiota.

Extensive reports have shown that SFB potently induces T_H_17/IL17A‐mediated immunity.^20^, ^21^, ^28^, ^29^, ^30^ IL17A signaling in stromal/osteoblastic cells critically regulates proinflammatory/pro‐osteoclastic cytokine expression,^14^, ^123^, ^124^ and CD4^+^ T_H_17 cell‐derived IL17A can have catabolic actions via signaling at stromal/osteoblastic cells.^14^, ^15^ Furthermore, IL17A has been reported to inhibit osteoblastogenesis.^125^, ^126^, ^127^ IL17A has also been shown to induce *Lcn2* in several cell types in vitro and in vivo.^124^, ^128^, ^129^, ^130^, ^131^ Therefore, SFB‐induced IL17A production in the gut may stimulate hepatic LCN2 synthesis and increased circulating levels of LCN2. Similar to IL17A, LCN2 has been shown to have a negative impact on bone formation and a positive effect on bone resorption.^88^, ^89^ Thus, increased circulating levels of IL17A and LCN2 are candidate mediators of SFB actions suppressing osteoblastogenesis, enhancing osteoclastogenesis, and impairing postpubertal skeletal growth and maturation. Recognizing that LCN2 and other hepatic innate immune serum factors are elevated in individuals afflicted by inflammatory bowel conditions,^132^, ^133^, ^134^ and that these individuals have an increased prevalence of osteopenia/osteoporosis,^135^, ^136^, ^137^ further underscores the relationship between the gut, liver, and bone.

Prior work has shown that SFB colonization can be modulated by diet,^138^, ^139^ probiotics,^140^ and antibiotics.^141^, ^142^, ^143^ This highlights the potential for the development of noninvasive clinical interventions in the pediatric gut microbiome, which could be utilized to support postpubertal skeletal growth and maturation. Although SFB colonizes humans and upregulates T_H_17 pathway genes,^24^, ^31^ it is important to note other commensals have been shown to clinically induce T_H_17/IL17A‐mediated immunity. *Bifidobacterium adolescentis*, a human gut commensal bacterium, promoted T_H_17 cell induction when colonized in mice.^144^ Prior studies have shown probiotics to have potential in limiting sex‐steroid deprivation‐associated bone loss in the aging skeleton.^145^, ^146^, ^147^, ^148^ Considering that probiotics can eradicate SFB colonization,^140^ our research suggests that this approach could be applied by pediatricians to optimize bone modeling and the attainment of peak bone mass in the growing skeleton. Appreciating that the late pubertal/adolescence phase accounts for roughly 40% of peak bone mass accrual,^39^, ^40^, ^41^, ^42^ therapeutic interventions supporting normal skeletal growth and maturation have lifelong implications for skeletal health and disease.

## Disclosures

All authors state that they have no conflicts of interest.

## Supporting information


**Figure S1**. Micro‐CT analyses of the tibia mid‐diaphysis cortical bone in GF versus SFB‐monoassociated mice (n = 5/gp). (*A*) Representative reconstructed cross‐sectional images of tibia mid‐diaphysis in GF and SFB‐monoassociated mice. (*B*) Cortical area fraction (Ct.Ar/Tt.Ar). (*C*) Cortical thickness (Ct.Th). Unpaired *t*‐test; data are presented as mean ± SEM.

**Figure S2**. Micro‐CT analyses of the tibia mid‐diaphysis cortical bone in EF and MPF mice (n = 4‐5/gp). (*A*) Representative reconstructed cross‐sectional images of tibia mid‐diaphysis in EF and MPF mice. (*B*) Cortical area fraction (Ct.Ar/Tt.Ar). (*C*) Cortical thickness (Ct.Th). Unpaired *t*‐test; data are presented as mean ± SEM.

**Figure S3**. (*A*–*C*) Nanostring analysis of acute‐phase reactant mRNA levels in livers of EF versus MPF mice (n = 5/gp). (*A*) *Crp* mRNA counts. (*B*) *Saa1* mRNA counts. (*C*) *Hamp* mRNA counts. (*D*) Long bone marrow, calvaria, tibia, and ileum were isolated from EF and MPF mice (n = 5/gp), and RNA was isolated for qRT‐PCR analysis of *Lcn2* mRNA. Relative quantification of mRNA was performed via the comparative *C*T method (2^‐ΔΔCT^). Unpaired *t*‐test; data are presented as mean ± SEM.

**Figure S4**. Long bone marrow, calvaria, and tibia were isolated from EF and MPF mice (n = 5/gp), and RNA was extracted for qRT‐PCR analysis of *Fgf2* mRNA. Relative quantification of mRNA was performed via the comparative *C*T method (2^−ΔΔCT^). Unpaired *t*‐test; data are presented as mean ± SEM.
