# Supplementary figures and images for: Specific Commensal Bacterium Critically Regulates Gut Microbiota Osteoimmunomodulatory Actions During Normal Postpubertal Skeletal Growth and Maturation
Source: JBMR Plus. 2020 Jan 30;4(3):e10338. doi: 10.1002/jbm4.10338 (PMC7059828; doi:10.1002/jbm4.10338)

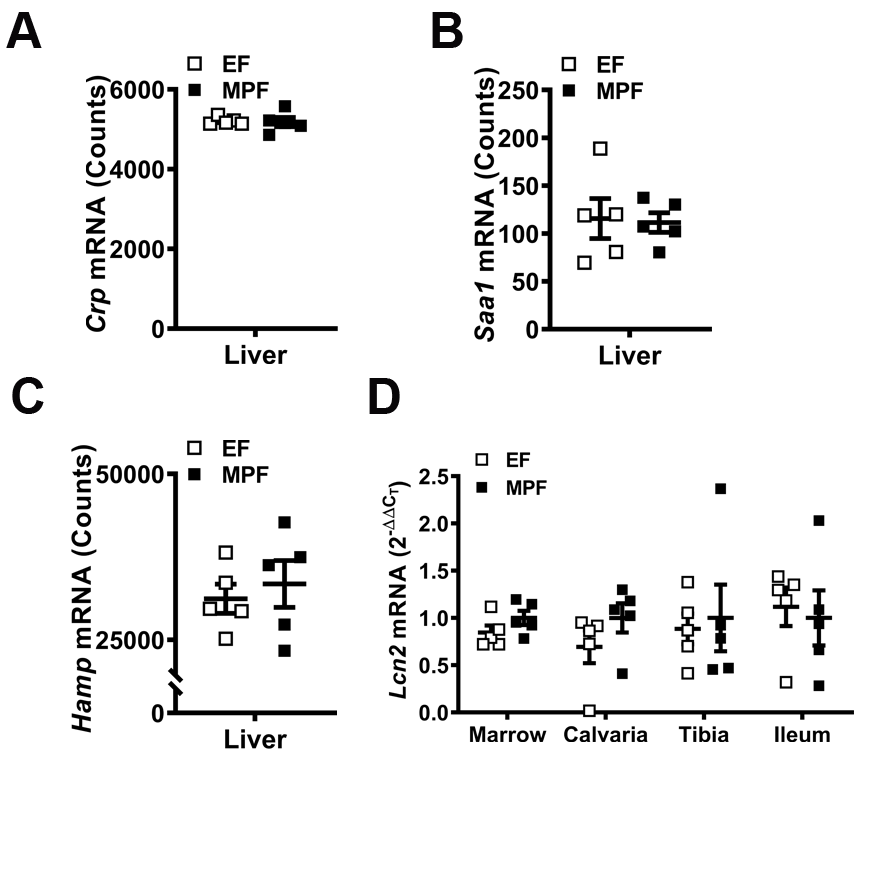

Supplement: Supplementary file 3 — Figure S3. (A–C) Nanostring analysis of acute‐phase reactant mRNA levels in livers of EF versus MPF mice (n = 5/gp). (A) Crp mRNA counts. (B) Saa1 mRNA counts. (C) Hamp mRNA counts. (D) Long bone marrow, calvaria, tibia, and ileum were isolated from EF and MPF mice (n = 5/gp), and RNA was isolated for qRT‐PCR analysis of Lcn2 mRNA. Relative quantification of mRNA was performed via the comparative CT method (2‐ΔΔCT). Unpaired t‐test; data are presented as mean ± SEM. [file JBM4-4-e10338-s003.tif]

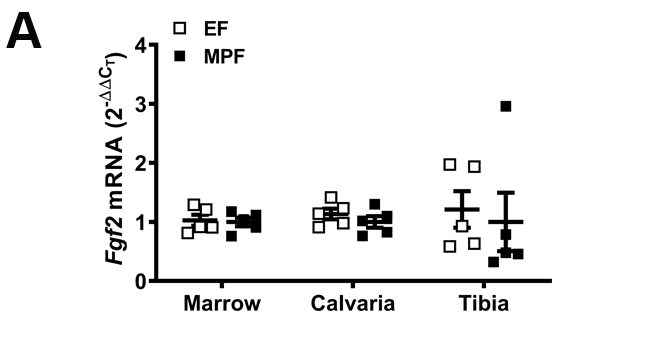

Supplement: Supplementary file 4 — Figure S4. Long bone marrow, calvaria, and tibia were isolated from EF and MPF mice (n = 5/gp), and RNA was extracted for qRT‐PCR analysis of Fgf2 mRNA. Relative quantification of mRNA was performed via the comparative CT method (2−ΔΔCT). Unpaired t‐test; data are presented as mean ± SEM. [file JBM4-4-e10338-s004.tif]
